# Genetic Analyses of Amphotericin B Susceptibility in *Aspergillus fumigatus*

**DOI:** 10.3390/jof7100860

**Published:** 2021-10-14

**Authors:** Yuying Fan, Gregory A. Korfanty, Jianping Xu

**Affiliations:** Department of Biology, McMaster University, Hamilton, ON L8S 4K1, Canada; fany8@mcmaster.ca (Y.F.); korfanga@mcmaster.ca (G.A.K.)

**Keywords:** aspergillosis, genome-wide association, minimum inhibitory concentration, genetic cross, PCR-RFLP, ascospores, SNP–SNP interaction, quantitative trait loci

## Abstract

*Aspergillus fumigatus* is a ubiquitous saprophytic mold that can cause a range of clinical syndromes, from allergic reactions to invasive infections. Amphotericin B (AMB) is a polyene antifungal drug that has been used to treat a broad range of systemic mycoses since 1958, including as a primary treatment option against invasive aspergillosis in regions with high rates (≥10%) of environmental triazole resistance. However, cases of AMB-resistant *A. fumigatus* strains have been increasingly documented over the years, and high resistance rates were recently reported in Brazil and Canada. The objective of this study is to identify candidate mutations associated with AMB susceptibility using a genome-wide association analysis of natural strains, and to further investigate a subset of the mutations in their putative associations with differences in AMB minimum inhibitory concentration (MIC) and in growths at different AMB concentrations through the analysis of progeny from a laboratory genetic cross. Together, our results identified a total of 34 candidate single-nucleotide polymorphisms (SNPs) associated with AMB MIC differences—comprising 18 intergenic variants, 14 missense variants, one synonymous variant, and one non-coding transcript variant. Importantly, progeny from the genetic cross allowed us to identify putative SNP–SNP interactions impacting progeny growth at different AMB concentrations.

## 1. Introduction

The fungal genus *Aspergillus* is one of the most well-studied fungal genera due to their medical, environmental, commercial, and industrial importance. *Aspergillus* species are ubiquitous in nature and can survive in a broad range of environmental conditions. Although there are over 350 identified *Aspergillus* species, only a few are pathogenic to humans [1]. Among these species, *Aspergillus fumigatus* is the most common cause of human *Aspergillus* infections, responsible for more than 90% of aspergillosis [1]. However, the frequency of aspergillosis caused by *A. fumigatus* varies among countries and patient groups [2]. Multiple physical characteristics of *A. fumigatus* allow the mold to be an efficient and widespread pathogen, resulting in the ubiquitous presence of up to tens of thousands of conidia/m^3^ of air [3]. Inhalation of these conidia can develop into aspergillosis. Although these spores can cause disease in healthy hosts, for the vast majority of immunocompetent individuals, they are quickly cleared by the innate immune system [4]. In hosts with a suppressed immune system, however, *A. fumigatus* can germinate, invade tissues through filamentous growth, and disseminate inside the host; resulting in the most severe presentation of aspergillosis, invasive aspergillosis [2]. It is estimated that over 300,000 cases of invasive aspergillosis occur annually, with ~10 million at risk [5]. The mortality rates associated with invasive aspergillosis range from 30 to 95% based on the patient population and underlying medical conditions [6]. However, the global burden of invasive aspergillosis is most likely underestimated due to reasons such as lack of surveillance measures and standardization, as well as the low sensitivity of current diagnostic assays [7,8].

For the treatment of aspergillosis, triazole drugs are recommended as first-line therapy. However, triazole-resistant *A. fumigatus* strains have been identified in six of the seven continents, with the presence of triazole-resistant strains reaching 80% in certain geographic and ecological populations [8,9,10,11,12,13,14,15,16,17,18,19,20,21,22]. Several factors have been identified as impacting the emergence and spread of triazole-resistant *A. fumigatus*, including the ecological source (environmental or clinical), underlying patient conditions, and agriculture fungicide use [13,23]. In addition, over the years, increased triazole resistance rates have been observed, e.g., 3.3% (2013) to 6.6% (2015) in Iran [24], 7.6% (2013) to 14.7% (2018) in the Netherlands [25], and 0.43% (1998–2011) to 2.2% (2015–2017) in the United Kingdom [26]. Patients with invasive aspergillosis caused by triazole-resistant *A. fumigatus* isolates have a high mortality rate, at ~88% [27]. In cases of infection by triazole-resistant isolates, amphotericin B (AMB) formulations have been recommended as the follow-up treatment of choice, and in cases of salvage therapy, particularly for refractory aspergillosis. In addition, AMB is suggested as the primary treatment in regions with ≥ 10% environmental triazole resistance rates [8,28].

AMB is a polyene drug that was introduced in the late 1950s and was the first antifungal agent used for treatment against invasive mycoses [29,30]. Despite 70 years of investigation and use, AMB’s mechanism(s) of action have not been fully elucidated and multiple models of action have been suggested. The majority of these models include the involvement of ergosterol, a major lipid component and the most abundant sterol found in fungal cell membranes [31]. The oldest and most accepted mechanism of action is the ion-channel model, wherein AMB binds to ergosterol and aggregates to form barrel-type pores in the fungal lipid bilayer [32]. These pores increase the permeability of the fungal cell membrane to K+ ions and other small cations, thereby allowing for the rapid depletion of intracellular ions that are vital for cell function [32]. The second model focuses on AMB’s ability to generate oxidative stress in cells by inducing the intracellular formation of reactive oxygen species (ROS) [32]. The accumulation of ROS causes oxidative damage to different macromolecules (lipids, proteins, and DNA). Although ROS are known to have a detrimental effect on fungal cells, their specific role in the fungicidal activity of AMB remains unknown. The third model involves surface absorption, in which AMB orients parallel to the membrane and sequesters ergosterol to the membrane surface, thus destabilizing the membrane [32]. The final model is known as the sterol sponge model, in which AMB primarily exists in the form of large extra-membranous aggregates that extract ergosterol from the lipid bilayer [32]. The diverse proposed modes of action for AMB underlie the complexity and multigenic nature of AMB susceptibility and resistance in *A. fumigatus*.

Despite over 60 years of clinical use, AMB is still widely used in medical therapy due to its broad spectrum of activity [30]. Furthermore, resistance to AMB, a fungicidal agent, is less common than resistance to fungistatic agents such as triazoles [33]. However, recent studies have identified high rates of AMB resistance in two geographic populations of *A. fumigatus*. In *A. fumigatus*, AMB-resistant strains are defined as having a minimum inhibitory concentration (MIC) greater than or equal to 2 mg/L. A study in Campinas, Brazil reported AMB resistance (MIC ≥ 2 mg/L) prevalence rates of 27% for *A. fumigatus* isolates and 43% in patients [34]. A high resistance (MIC ≥ 2 mg/L) rate of 96.4% was also reported in Hamilton, Canada, and this is the highest reported rate to date [35]. At present, the reasons behind the emergence of high AMB resistance rates in these two geographic populations are unknown. Moreover, the proposed mechanisms for AMB resistance in *A. fumigatus* have mostly come from studies on human pathogenic and non-pathogenic yeasts. In studies of drug resistance among human fungal pathogens, species often differ in their intrinsic drug susceptibility patterns, and possess species-specific mechanisms for drug resistance. Thus, it is important to understand the mechanisms of resistance for individual species. Currently, there is little information available about the mechanism(s) of AMB resistance in *A. fumigatus*, and mutations that confer resistance remain largely unexplored.

In our recent paper, we investigated 71 *A. fumigatus* isolate genomes with known AMB MIC values, in order to examine non-synonymous mutations in 22 genes of interest potentially associated with AMB susceptibility. We also conducted a genome-wide association study (GWAS) on the clade level using 33 strains [36]. Those 22 genes of interest included those involved in ergosterol biosynthesis, ROS detoxification, and the high-osmolarity glycerol mitogen-activated protein kinase pathway. In total, we identified over 60 candidate single-nucleotide polymorphisms (SNPs) associated with AMB resistance [36]. In that study, strains were classified into two binary classes for analyses, AMB susceptible vs. resistant. The objective of this paper is to expand previous investigations by using more samples and focusing on the quantitative nature of AMB susceptibility. Specifically, we aimed to identify the genetic variations associated with differences in AMB MIC in *A. fumigatus* by conducting a GWAS using a larger sample set of 98 *A. fumigatus* strains. A subset of these identified mutations was then examined for their associations with specific AMB MIC values and growths at different AMB concentrations among progeny strains from a defined genetic cross. Specifically, we mated two AMB-resistant strains, CM11 (MIC = 8 mg/L) from Hamilton, Ontario and the supermater AFB62-1 (MIC = 4 mg/L), which were known to differ at five SNPs identified as associated with AMB susceptibility in GWAS. The progeny strains were used to examine the contributions of these SNP sites to the observed differences in AMB MIC values and in fungal growths at different AMB concentrations. To help readers follow the paper, we have listed the common acronyms and abbreviations used throughout in Appendix A.

## 2. Materials and Methods

### 2.1. Whole-Genome Sequences and Variant Calling

A total of 98 *A. fumigatus* whole-genome sequences were used in this study, of which 86 sequences were downloaded from the National Center for Biotechnology Information (NCBI) Sequence Read Archive and the remaining 12 sequences were obtained from our previous study [36]. The strain sample set was collected from 9 countries, which consisted of 10 strains from Canada, 5 strains from Germany, 7 strains from India, 1 strain from Ireland, 31 strains from Japan, 10 strains from the Netherlands, 18 strains from Spain, 11 strains from the United Kingdom, and 5 strains from the United States. The geographical location, source, AMB MIC values and genome sequence accession numbers for all 98 strains are listed in Supplementary Table S1.

Sequence mapping, assembly and variant calling were performed using the same pipeline reported in our previous study for triazole GWAS [37]. Briefly, read quality was checked with FastQC v0.11.5 and trimmed using Trimmomatic v0.36 [38]. Reads were mapped and aligned using the *A. fumigatus* reference genome Af293 (GenBank accession GCA_000002655.1) via the BWA-MEM algorithm v0.7.17 [39]. The MarkDuplicates (Picard) tool was used to identify and remove duplicate reads. Variant calling was performed using FreeBayes v0.9.21-19 [40] and variant filtering using vcftools [41] to remove indels, variants with a quality score below 15, and variants with a call rate less than 0.90. A second filtering step was carried out using vcftools to remove multiallelic sites. The resulting filtered VCF file was denoted as the “soft-filtered” file and contained 277,669 SNP sites. Variant annotation and functional effect predictions were performed using SnpEff v5.0 and the reference genome Af293 [42]. Variant pruning was conducted using PLINK 1.90 beta to remove highly linked variants (VIF > 2) [43]. 

### 2.2. Genome-Wide Association Study and Linkage Disequilibrium

Association analysis was performed in TASSEL 5 by implementing the mixed linear model approach, which handles both fixed and random effects in the model. The analysis included a population structure defined by 5 principal component vectors, determined based on the scree plot, and a kinship matrix calculated using the Identity by State (Centered IBS) method to account for cryptic relatedness as a random effect [44]. To avoid biases in the association analysis due to imbalanced allele frequencies, a minor allele frequency threshold of 0.05 was set using TASSEL 5. A total of 20,929 SNP sites were retained and used in the AMB association analysis. Linkage disequilibrium analysis was also conducted on the resulting 20 SNPs with the lowest *p*-values and all 277,669 SNP sites from the soft-filtered file were then used to identify highly linked (R^2^ > 0.85) SNPs of interest.

### 2.3. Mating and Ascospore Collection

A genetic cross was created between two *A. fumigatus* strains, CM11 and AFB62-1. CM11 had an AMB MIC of 8 mg/L and, to our knowledge, this is the highest reported AMB MIC in *A. fumigatus*. CM11 has the MAT1-2 mating type. AFB62-1 had an AMB MIC of 4 mg/L and is the designated supermater with mating type MAT1-1, capable of mating with many strains of MAT1-2 mating type to complete the sexual cycle in a relatively short period of time [45].

The mating and harvesting of *A. fumigatus* cleistothecia was conducted using a modified protocol from Ashton and Dyer [46]. The cross was conducted on oatmeal agar medium, sealed with parafilm, wrapped in aluminum foil, and incubated inverted at 30 °C. After one month, single ascospore progenies were harvested from the cleistothecium. Underneath a dissecting microscope, single cleistothecia were isolated using a fine-point sterile syringe. The cleistothecia were washed from any adhering conidia by rolling them on a 4% water agar medium. Two washed cleistothecia were then placed in 0.01% TWEEN 20 solution and crushed using a fine-point sterile syringe to release the ascospores. The solution was vortexed to ensure the cleistothecia had been sufficiently broken and all ascospores were released. Using a hemocytometer, the ascospore solutions were adjusted to a concentration of ~2.00 × 10^3^ CFU/mL using TWEEN 20. The solutions underwent heat treatment at 70 °C for 1 h to kill any remaining conidia, then 100 μL of the ascospore suspension was plated on malt agar plates and incubated at 30 °C for 2 to 3 days. After incubation, single ascospore-derived colonies were picked using a sterile loop and each was transferred to new medium for phenotypic and genotypic analyses, as described below.

### 2.4. AMB Susceptibility Testing 

The in vitro susceptibility of all sexual progeny and the two parental strains was determined using the M38-A2 guideline of the Clinical and Laboratory Standards Institute (CLSI) [47]. Briefly, strains were grown on Sabouraud dextrose agar for 48 h at 37 °C. The asexual spores, conidia, were harvested from each strain and spore suspensions were adjusted to an optical density at 530 nm from 0.09 to 0.13. Using the RPMI-1640 medium, a 1:50 dilution was produced to obtain a final concentration of ~0.4 × 10^5^ to 5 × 10^6^ CFU/mL. Spore suspensions were placed into 96-well microtiter plates containing varying concentrations of AMB and incubated at 35 °C for 48 h. The AMB concentrations tested were 0 mg/L, 0.25 mg/L, 0.5 mg/L, 1 mg/L, 2 mg/L, 4 mg/L, 8 mg/L, and 16 mg/L. *Candida parapsilosis* (ATCC 22019) and *Candida krusei* (ATCC 6258) were used as quality controls. The AMB MIC of all progeny and parental strains were determined based on the procedures as recommended by M38-A2. In addition, the amount of growth at each drug concentration for all strains was measured spectrophotometrically at 530 nm. The ratio of fungal growth for strains at various AMB concentrations was calculated by comparing the optical density measurements at 530 nm (OD_530_) at the start of incubation (0 h) and at the end of incubation (48 h). The value difference between the two time points compared to the positive control (0 mg/L AMB) was taken as the rate of fungal growth over this time period for each AMB concentration. Antifungal susceptibility testing was performed with three replicates. Outlying absorbance values were assessed and removed using a Dixon’s Q-test (α = 0.1). The mean value of three technical repeats was taken to determine the rate of fungal growth for each strain at each AMB concentration. 

### 2.5. DNA Extraction of the Progeny Strains

DNA extraction of the progeny and parental strains was performed using a modified protocol described by Xu and colleagues [48]. Conidia were grown in 1 mL of Sabouraud dextrose broth for 48 h at 37 °C. After incubation, the tubes were centrifuged at 13,000× *g* rpm for 10 min and the supernatant was discarded. The cells were resuspended in 0.5 mL of protoplasting buffer and incubated at 37 °C for 2 h. The solutions were then centrifuged at 5000× *g* rpm for 10 min. The supernatant was poured out and 0.5 mL of lysing buffer was added in. The mixture was vortexed and incubated at 65 °C for 30 min, and 500 µL of chloroform/isoamyl alcohol (24:1) and 125 µL of 7.5 M ammonium acetate was added to each sample. The tubes were vortexed and centrifuged at 13,000× *g* rpm for 15 min, or until the upper layer was clear; 500 µL from this clear layer was added to 550 µL of ice-cold isopropyl alcohol. The tubes were mixed by inversion, centrifuged at 13,000× *g* rpm for 2 min, and the remaining supernatant was discarded. DNA pellets were washed using 50 µL of 70% ethanol for 2 min, dried overnight, and resuspended in 60 µL of 1× TE buffer. 

### 2.6. Polymerase Chain Reaction and Restriction Fragment Length Polymorphism

The progeny genotypes at five SNP sites were determined using polymerase chain reaction (PCR) and restriction fragment length polymorphism (RFLP) (PCR-RFLP). The details for the five SNPs can be found below in the Results section. Among the five SNP sites, four were located on chromosome 5 and one on chromosome 6. Primers flanking the SNP sites were designed using the whole-genome sequences of CM11 and AFB62-1. PCR amplification was conducted using a SimpliAmp Thermal Cycler and PCR products were checked using 1% agarose gels. Restriction digests that distinguish nucleotide bases at the five SNP sites between the two parents were performed on all progeny strains, following the manufacturer’s instructions (NEB, UK). The digested products were run on 2% agarose gels at 80 V for 1.5 h. Progeny with a PCR-RFLP pattern identical to one of the two parents at each locus were scored as having the allele (nucleotide) of the specific parent at the specific SNP position. Information on the primer sequences, PCR amplification conditions, and restriction enzymes can be found in Table 1.

## 3. Results

### 3.1. Genome-Wide Association Study and Linkage Disequilibrium Analysis

**Figure 1:**
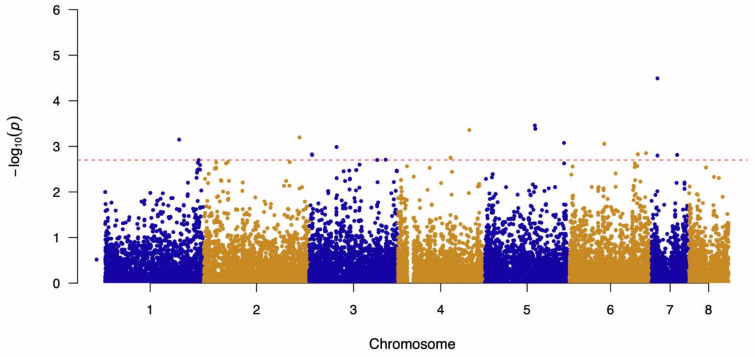
Manhattan plot based on the GWAS results for SNPs associated with Amphotericin B sensitivity in A. fumigatus. The red dashed line indicates the separation for the top 20 SNPs.

A genome-wide association study (GWAS) was conducted to determine candidate mutations associated with AMB susceptibility using a total of 98 A. fumigatus whole-genome sequences and their corresponding AMB MIC values. The results of the GWAS are presented in a Manhattan plot (Figure 1). The quantile–quantile plot of observed and expected *p*-values showed no genomic inflation (Supplementary Figure S1). 

From the GWAS results, the top 20 significant SNPs with the smallest *p*-values were further examined. Among these 20 SNPs, 13 (65%) were located in intergenic regions, 6 (30%) were missense variants and 1 (5%) was a synonymous variant (Table 2).

Using the top 20 SNPs and all 277,669 variants from the soft-filtered file, linkage disequilibrium analysis was conducted to identify highly linked (R^2^ > 0.85) SNPs of interest. From this analysis, 24 highly linked variants were found (Table 3). The additional 24 variants consisted of 17 intergenic variants, four missense variants, one synonymous variant and two non-coding transcript variants (Table 3). Fisher’s exact tests were further conducted on these 24 highly linked variants to determine SNPs significantly associated with AMB resistance. The results are summarized in Table 3.

In our previous GWAS of AMB resistance, a total of 71 A. fumigatus strains were analyzed. Through the use of Fisher’s exact tests, 12 missense variants were found to be significantly associated with AMB resistance using an uncorrected *p*-value significance threshold of 0.05 [36]. These 12 SNPs were located in six genes of interest: *erg3* (*n* = 2), *tcsB* (*n* = 4), *mpkC* (*n* = 2), *catA* (*n* = 2), *fos1* (*n* = 1), and *mpkB* (*n* = 1). These SNP sites were also examined in our current study using the expanded 98-strain sample set and via Fisher’s exact tests using a Bonferroni-corrected *p*-value significance threshold. The results of these tests are shown in Table 4. 

In this test, the European Committee on Antimicrobial Susceptibility Testing (EUCAST) MIC breakpoint of >1 mg/L was used to define AMB-resistant A. fumigatus strains [49]. From the Fisher’s tests and using a Bonferroni-corrected significance threshold of 1.39 × 10^−3^ (0.05/36), 8 of the 24 highly linked SNPs identified in the current analyses were significantly associated with AMB resistance (Table 3). Among these eight SNPs, four were on chromosome 4 and were intergenic variants found between *AFUA_4G09240* and *AFUA_4G09250*. The remaining four SNPs were located on chromosome 5: two were missense variants in *AFUA_5G00710* and in *AFUA_5G09220*, one was a non-coding transcript variant in *AFUA_5G09320*, and the final SNP was found in the intergenic region between *AFUA_5G00700* and *AFUA_5G00710* (Table 3). The Fisher’s exact tests for the previous 12 missense variants of interest found six missense variants significantly associated with AMB resistance in the current sample set (Table 4). These six SNPs were found in three genes and comprised missense variants in the three genes *tcsB* (*n* = 3 SNPs), *mpkC* (*n* = 2 SNPs), and *catA* (*n* = 1 SNP) (Table 4).

### 3.2. Mating Cross and AMB Susceptibility of Progeny

To further confirm the genetic association between the candidate mutations of interest identified above with AMB susceptibility, we investigated the genotype–phenotype associations among progeny strains of the mating cross. From the mating cross between CM11 and AFB62-1, we obtained 143 meiotic progenies. The AMB MIC values for the 143 progeny strains and the two parental strains are listed in Supplementary Table S2. The parental strains CM11 and AFB62-1 had an AMB MIC of 8 mg/L and 4 mg/L, respectively. Among the 143 progeny strains, 4 (2.80%) strains had an MIC value of 2 mg/L, 120 (83.92%) strains had an MIC of 4 mg/L, and the remaining 19 (13.29%) strains had an MIC of 8 mg/L (Supplementary Table S2). The generation of a novel MIC class of 2 mg/L in the progeny population is consistent with the two parental strains having different genetic mechanisms contributing to AMB resistance.

**Figure 2:**
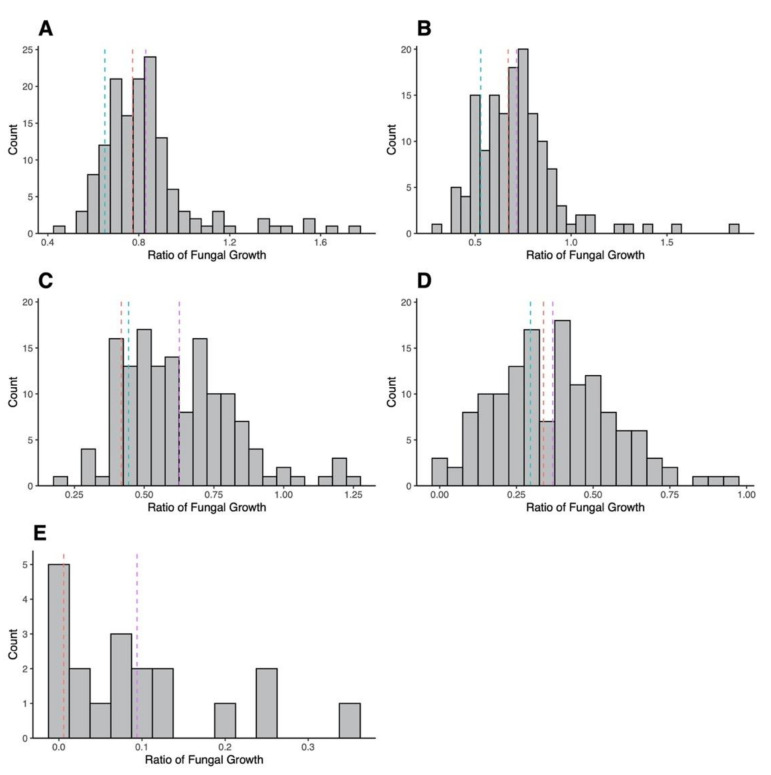
Distribution of growth ratio values for the progeny strains measured at Amphotericin B concentrations of (**A**) 0.25 mg/L (*n* = 143), (**B**) 0.50 mg/L (*n* = 143), (**C**) 1.00 mg/L (*n* = 143), (**D**) 2.00 mg/L (*n* = 139), and (**E**) 4.00 mg/L (*n* = 19). Fungal growth was determined by calculating difference in OD_530_ at start (0 h) and end of incubation (48 h); this value was divided by fungal growth in the positive control (0 mg/L) to determine the ratio of fungal growth. Dashed lines represent the values of the two parental strains, CM11 (red) and AFB62-1 (blue), as well as the mean value for the progeny (purple).

In addition to MIC values, the growth of the progeny strains in various concentrations of AMB was also determined as another quantitative measure of AMB susceptibility. The amounts of fungal growth for the 143 progeny strains and parental strains in the varying concentrations of AMB (0.25 mg/L, 0.50 mg/L, 1.00 mg/L, 2.00 mg/L and 4.00 mg/L) were measured using spectrophotometry (Supplementary Table S2). The distribution of growth ratio values for all 145 strains can be found in Figure 2. 

The growth data shown in Figure 2 indicate the large quantitative variation in progeny growth at different AMB concentrations. Interestingly, we found abundant evidence for transgressive phenotypes in the progeny population in both directions at all five AMB concentrations (Figure 2). At an AMB concentration of 0.25 mg/L, 83 (58.04%) progeny strains had a higher amount of fungal growth than both parents, while 16 (11.19%) progeny had lower growths than both parents (Supplementary Table S2). Furthermore, four (2.80%) progeny strains also had growth values more than two-fold higher than the faster parent, and no progeny had values two-fold lower than the slower parent. At the 0.50 mg/L concentration, 81 (56.64%) progeny strains had higher fungal growths than both parents, while 26 (18.18%) progeny had lower growth. Three (2.10%) strains also had growth values more than two-fold higher than the faster parent and no strains had values two-fold lower than the slower parent. At a concentration of 1.00 mg/L, 118 (82.52%) strains had higher growths than both parents while 18 (12.59%) had lower values than both. Twelve (8.39%) strains had values more than two-fold higher than the faster parent and one (0.70%) strain had a growth value more than two-fold lower than the slower parent. At a concentration of 2.00 mg/L, 73 (52.52%) strains had growths higher than the parental strains, while 58 (41.73%) had values lower than both parents. In addition, 8 (5.76%) strains had values more than two-fold higher than the faster parent and 17 (12.23%) strains had values more than two-fold lower than the slower parent. At the final concentration of 4.00 mg/L, 16 (84.21%) strains had growth values higher than the CM11 parent and 3 (15.79%) strains had values lower than CM11. Fourteen (73.68%) strains had growth values more than two-fold higher than CM11 and no strains had values more than two-fold lower than CM11 (Supplementary Table S2). Together, these results indicate a substantial difference between the two parental strains in the genetic mechanisms of AMB MIC.

**Figure 3:**
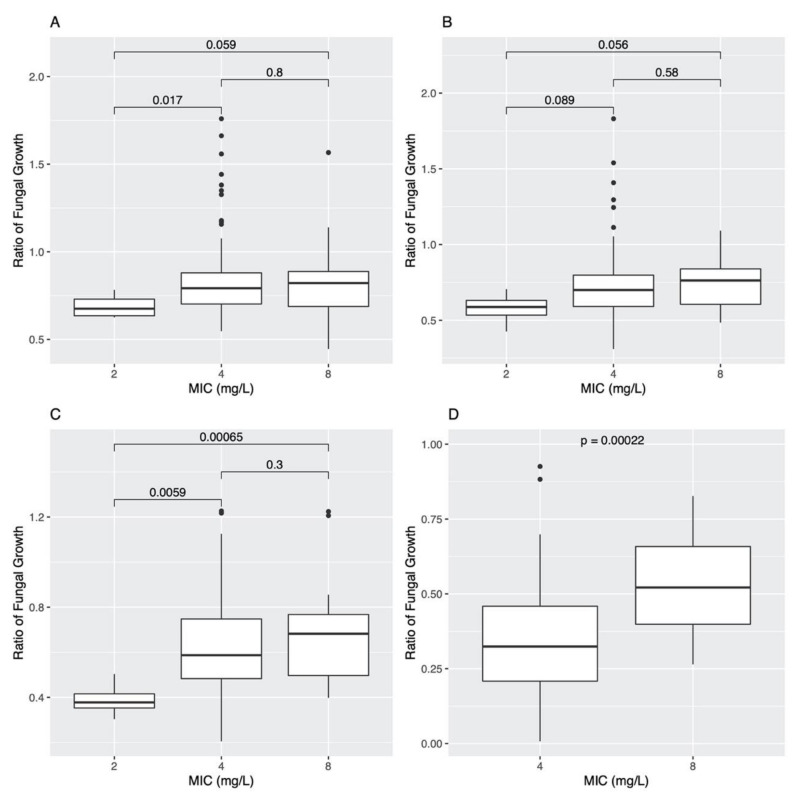
Ratio of fungal growth for the 143 progeny strains in Amphotericin B concentrations of (**A**) 0.25 mg/L, (**B**) 0.50 mg/L, (**C**) 1.00 mg/L, and (**D**) 2.00 mg/L. Fungal growth was determined by calculating difference in OD_530_ at start (0 h) and end of incubation (48 h); this value was divided by fungal growth in the positive control to determine ratio of fungal growth. Welch’s *t*-test *p*-values are also denoted to compare the AMB MIC groups of 2 mg/L (*n* = 4), 4 mg/L (*n* = 120), and 8 mg/L (*n* = 19). Differences were considered statistically significant at *p* < 0.05.

At each AMB concentration, Welch’s t-tests were also conducted on the progeny strains to compare the ratio of fungal growth and AMB MIC values (Figure 3). 

The results of these tests were that statistically significant differences (*p* < 0.05) between MIC groups were present at AMB concentrations of 0.25 mg/L, 1.00 mg/L and 2.00 mg/L (Figure 3A,C,D). The only exception was at the AMB concentration 0.50 mg/L, where no statistically significant differences in growths were present between MIC groups (Figure 3B). At 0.25 mg/L, fungal growths were statistically significantly higher in the 4.00 mg/L MIC progeny group compared to the 2 mg/L MIC progeny group (*p* = 0.017; Figure 3A). At the concentration of 1.00 mg/L, the mean growth of the 2 mg/L MIC progeny group was significantly lower than that of both the 4 mg/L and 8 mg/L MIC progeny groups, at *p* = 0.0059 and *p* = 0.00065, respectively (Figure 3C). Lastly, at the 2.00 mg/L concentration, the mean growth of the 8 mg/L MIC progeny group was statistically significantly higher than that of the 4 mg/L MIC progeny group; *p* = 0.00022 (Figure 3D). These results suggest that strains with higher AMB MICs typically grow faster than those with low AMB MICs at various AMB concentrations below their MIC.

### 3.3. Progeny Genotyping

From our final 20,929 SNP sites and using a pairwise SNP comparison, 3960 SNPs were found between the two parental strains AFB62-1 and CM11. For the progeny genotype analyses, we focused on the top 20 SNP sites obtained from the AMB GWAS and the 8 putatively associated highly linked SNPs obtained from the linkage disequilibrium analysis (Supplementary Table S3). From the 28 SNPs, 5 were selected for further investigation in the 143 progeny strains (Table 5). Given that these two AMB-resistant strains were different at these five SNPs (and many other SNPs), we hypothesized that these SNPs were either false positives from GWAS, or that their contributions to AMB susceptibility would likely be quantitative, potentially influencing the growth differences between these two strains at various AMB concentrations. Significantly, alternative alleles at these five SNPs could be readily distinguished by restriction fragment length polymorphisms, either directly at the SNP site (for three SNPs, SNP 1, SNP 3, and SNP 4) or at a close-by SNP site within 1000 bp of the AMB susceptibility SNPs identified by GWAS (for two SNPs, SNP 2 and SNP 5, using a representative SNP site 656 bp downstream and 723 bp downstream, respectively, for genotyping). The five SNP sites comprised three intergenic variants, one missense variant and one non-coding transcript variant (Table 5). The detailed progeny genotypes at these five SNP sites were determined using PCR-RFLP analysis and are presented in Supplementary Table S2. 

### 3.4. Association between Variant SNPs and AMB MIC and Growths at Different AMB Concentrations among Sexual Progeny Strains

#### 3.4.1. Analyses Based on Individual SNPs

For each of the five SNP sites, Fisher’s exact tests were conducted between the progeny AMB MIC and the inherited parental allele (Table 6). Using a Bonferroni-corrected *p*-value threshold of 0.01 (0.05/5), no statistically significant differences were observed between the MIC groups in their frequencies of inherited alleles at any of the five SNPs (Table 6).

Similar to the observed lack of statistically significant differences between individual SNPs and the MIC groups, we observed the limited contribution of these five SNPs individually to growth differences among progeny at the tested AMB concentrations. Specifically, for each of the five SNP sites, Welch’s *t*-tests were conducted to compare the ratios of fungal growth at each AMB concentration between the progeny genotypes (Supplementary Figure S2). Based on these tests, a statistically significant difference (*p* = 0.047) was found at an AMB concentration of 0.25 mg/L for SNP site 5. Specifically, progeny that inherited the CM11 allele at SNP 5 showed statistically significantly higher fungal growth than progeny that inherited the AFB62-1 allele (Supplementary Figure S2). No difference was observed at the other four SNP sites.

#### 3.4.2. Analyses Based on Pairs of SNP Combinations

To analyze the effects of SNP–SNP interactions on differences in AMB MIC and fungal growth among progeny, all possible pairwise SNP combinations between these five sites were also assessed. In terms of MIC values, Fisher’s exact tests were conducted among the three MIC groups (MIC = 2 mg/L, 4 mg/L, or 8 mg/L) and the pairwise SNP combinations. No statistically significant difference was found between the genotype groups in their AMB MIC values using the Bonferroni-corrected *p*-value threshold of 0.005 (0.05/10) (Table 7).

**Figure 4:**
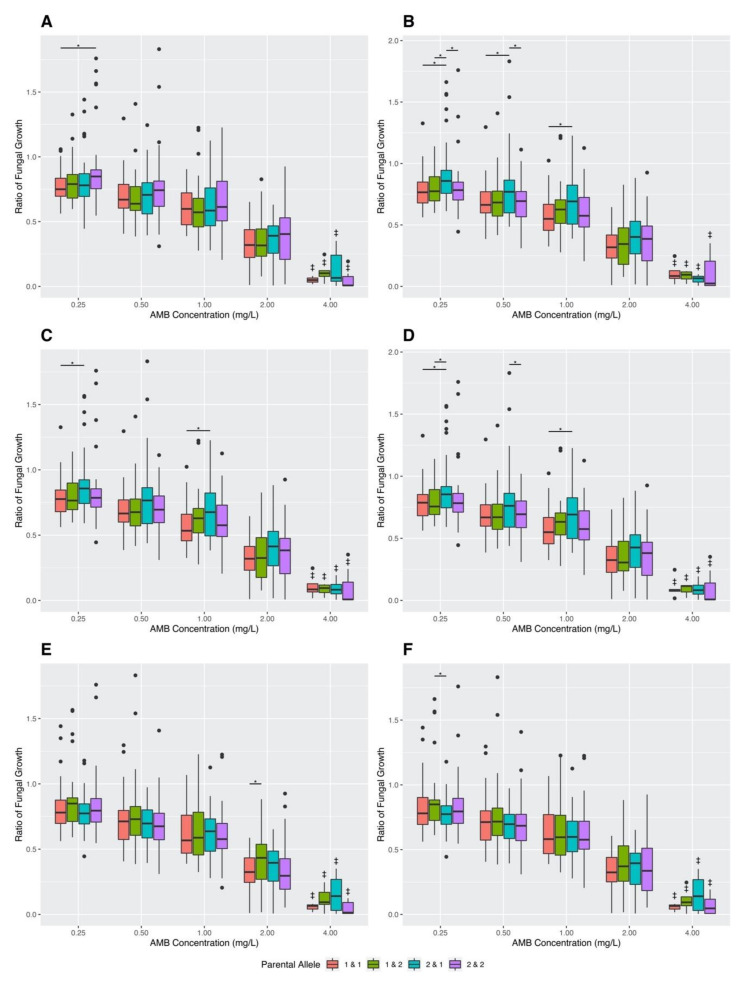
Growths of the 143 progeny strains in varying Amphotericin B concentrations, grouped based on pairwise variant genotype at (**A**) SNP 5 and 1, (**B**) SNP 5 and 2, (**C**) SNP 5 and 3, (**D**) SNP 5 and 4, (**E**) SNP 4 and 1, and (**F**) SNP 2 and 1. Fungal growth was determined by calculating differences in OD_530_ at start (0 h) and end of incubation (48 h); this value was divided by fungal growth in the positive control to determine ratio of fungal growth. Parental allele 1 denotes the AFB62-1 genotype and parental allele 2 denotes the CM11 genotype. “*” denotes statistically significant differences (Welch’s *t*-test *p*-values < 0.05) and “^‡^” indicates bar groups with *n* ≤ 12.

In addition to examining MIC values, Welch’s *t*-tests were again conducted using the pairwise genotype combinations to compare the ratios of fungal growth in varying AMB concentrations (Figure 4). The *p*-values for all conducted Welch’s *t*-tests of the 10 pairwise SNP combinations can be found in Supplementary Figure S3. The results of this analysis showed statistically significant differences in fungal growth ratios for six of the 10 pairwise combinations: SNP 5 and 1, SNP 5 and 2, SNP 5 and 3, SNP 5 and 4, SNP 4 and 1, and SNP 2 and 1 (Figure 4). Below we describe the effects for each of the six significant SNP–SNP combinations.

For the pairwise combination of SNP 5 and 1, a statistically significant difference was found at an AMB concentration of 0.25 mg/L. Here, progeny strains that inherited the variant alleles from CM11 at both SNP sites had a higher mean fungal growth ratio than progeny strains that inherited both variant genotypes from AFB61-2 (Figure 4A). 

For the pairwise combination of SNP 5 and 2, statistically significant differences were found at three AMB concentrations of 0.25 mg/L, 0.50 mg/L, and 1.00 mg/L (Figure 4B). At a concentration of 0.25 mg/L, progeny that inherited the CM11 genotype at SNP 5 and the AFB62-1 genotype at SNP 2 had higher mean fungal growth ratios than progeny that inherited the AFB62-1 genotype at both SNP sites, progeny that inherited the AFB62-1 genotype at SNP 5 and the CM11 genotype at SNP 2, and progeny that inherited the CM11 genotype at both SNP sites. At a concentration of 0.50 mg/L, progeny that inherited the CM11 genotype at SNP 5 and the AFB62-1 genotype at SNP 2 had higher mean fungal growths than progeny that inherited the AFB62-1 genotype at both SNP sites, and progeny that inherited the CM11 genotype at both SNP sites. Lastly, at an AMB concentration of 1.00 mg/L, progeny that inherited the CM11 genotype at SNP 5 and the AFB62-1 genotype at SNP 2 had higher mean fungal growth ratios than progeny that inherited the AFB62-1 genotype at both SNP sites (Figure 4B). 

For the pairwise combination of SNP 5 and 3, statistically significant differences were found at AMB concentrations of 0.25 mg/L and 1.00 mg/L (Figure 4C). At both AMB concentrations of 0.25 mg/L and 1.00 mg/L, progeny that inherited the CM11 genotype at SNP 5 and the AFB62-1 genotype at SNP 3 had higher mean fungal growths than progeny that inherited the AFB62-1 genotype at both SNP sites (Figure 4C). 

For the pairwise combination of SNP 5 and 4, statistically significant differences were found at AMB concentrations of 0.25 mg/L, 0.50 mg/L, and 1.00 mg/L (Figure 4D). At 0.25 mg/L, progeny that inherited the CM11 genotype at SNP 5 and the AFB62-1 genotype at SNP 4 had higher mean fungal growths than progeny that inherited the AFB62-1 genotype at both SNP sites, and progeny that inherited the AFB62-1 genotype at SNP 5 and CM11 genotype at SNP 4. At an AMB concentration of 0.50 mg/L, progeny that inherited the CM11 genotype at SNP 5 and the AFB62-1 genotype at SNP 4 had higher mean fungal growths than progeny that inherited the CM11 genotype at both SNP sites. Lastly, at an AMB concentration of 1.00 mg/L, progeny that inherited the CM11 genotype at SNP 5 and the AFB62-1 genotype at SNP 4 had higher mean fungal growth ratios than progeny that inherited the AFB62-1 genotype at both SNP sites (Figure 4D).

For the pairwise combination of SNP 4 and 1, statistically significant differences were found at the AMB concentration of 2.00 mg/L (Figure 4E). Progeny strains that had the AFB62-1 genotype at SNP 4 and the CM11 genotype at SNP 1 had a higher mean fungal growth than progeny strains that inherited both variant genotypes from CM11 (Figure 4E). 

Finally, for the pairwise combination of SNP 2 and 1, statistically significant differences were found at the AMB concentration of 0.25 mg/L (Figure 4F). Progeny strains with the AFB62-1 genotype at SNP 2 and the CM11 genotype at SNP 1 had a higher mean fungal growth than those with the CM11 genotype at SNP 2 and AFB62-1 at SNP 1 (Figure 4F).

#### 3.4.3. Analyses Based on Linked SNPs

**Figure 5:**
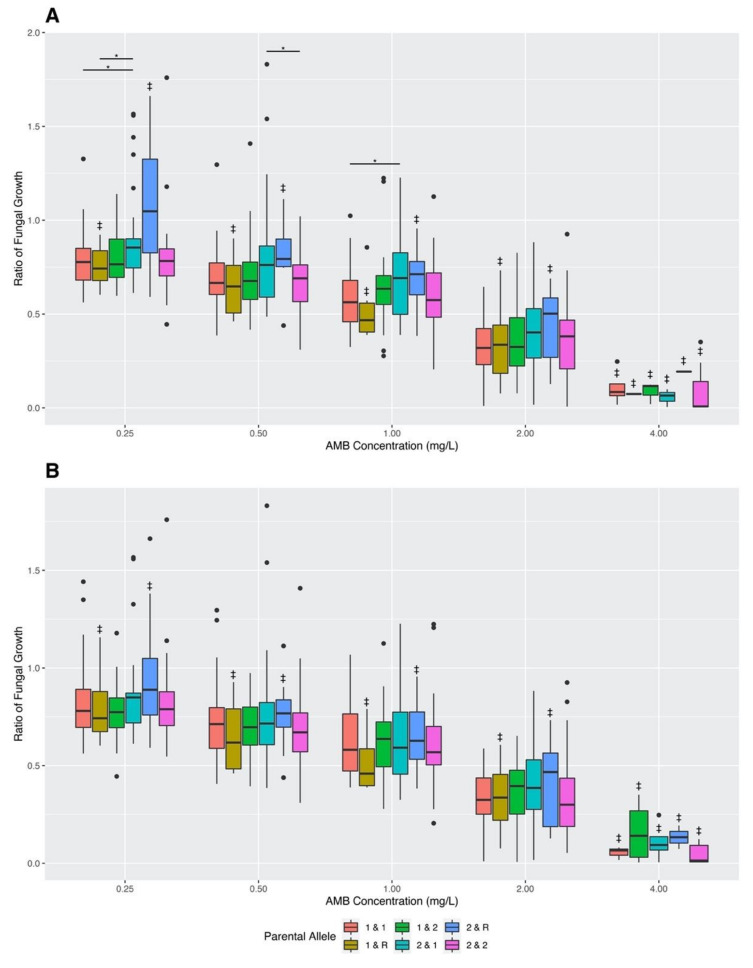
Growths of the 143 progeny strains in varying Amphotericin B concentrations, grouped based on the variant genotype combination at the sites (**A**) SNP 5 and Group A and (**B**) SNP 1 and Group A. Fungal growth was determined by calculating difference in OD_530_ at start (0 h) and end of incubation (48 h); this value was divided by fungal growth in the positive control to determine ratio of fungal growth. Parental allele 1 denotes the AFB62-1 genotype, parental allele 2 denotes the CM11 genotype, and R denotes recombination in the SNP group. “*” denotes statistically significant differences (Welch’s *t*-test *p*-values < 0.05) and “^‡^” indicates bar groups with *n* ≤ 12.

Additionally, a subset of these five SNP sites showed low rates of recombination in the progeny. The group, denoted as Group A, consisted of SNP 2, SNP 3, and SNP 4. Among the 143 progeny strains, 64 (44.76%) strains inherited all three genotypes from AFB62-1, 67 (46.85%) strains inherited all three genotypes from CM11, and 12 (8.39%) strains had recombinations present at these three sites (Supplementary Table S2). Using this additional grouping, Welch’s t-tests were performed for the additional combinations of SNP 5 and Group A, and SNP 1 and Group A (Figure 5). 

The additional analyses of SNP 5 and Group A found statistically significant differences present at AMB concentrations of 0.25 mg/L, 0.50 mg/L and 1.00 mg/L (Figure 5A). At the AMB concentration of 0.25 mg/L, progeny that inherited the CM11 genotype at SNP 5 and the AFB62-1 genotype for all Group A SNP sites had a statistically higher mean fungal growth than progeny with the AFB62-1 genotype at all four SNP sites, and progeny with the AFB62-1 genotype at SNP 5 and a recombination present in Group A. At 0.50 mg/L, progeny with the CM11 genotype at SNP 5 and the AFB62-1 genotype for all Group A sites had a higher mean fungal growth than progeny with the CM11 genotype at all four SNP sites. Lastly, at the AMB concentration of 1.00 mg/L, progeny with the CM11 genotype at SNP 5 and the AFB62-1 genotype at Group A SNP sites had a higher mean fungal growth than progeny with the AFB62-1 genotype at all four SNP sites (Figure 5A). For the SNP 1 and Group A combination, no statistically significant differences were present at any AMB concentration (Figure 5B). 

Together, both the pairwise SNP–SNP interaction analyses and the linked SNP analyses revealed that many recombinant genotypes at these five SNP sites showed greater growths than either parental genotype. The results are consistent with the two parental strains having different genetic mechanisms controlling AMB susceptibility, and suggest that AMB susceptibility is a quantitative and multigenic trait.

## 4. Discussion

In this study, our combined GWAS and genetic crossing approaches revealed that multiple genes contribute to differences in AMB MICs and in fungal growths among strains at different AMB concentrations in *A. fumigatus*. The GWAS was conducted using 98 *A. fumigatus* whole-genome sequences from strains across nine countries with reported AMB MIC values ranging from 0.06 to 8 mg/L. From the GWAS analysis, among the top 20 SNPs, 6 were missense variants. The six missense variants were located in six genes. The highest scoring missense variant was found in *AFUA_4G12480*, which encodes for an asparagine synthase that converts aspartate to asparagine in an ATP-dependent reaction [50]. The second highest scoring missense variant was in *AFUA_6G12420*, a putative SprT family metallopeptidase. The next two missense variants were found in the uncharacterized proteins *AFUA_6G12460* and *AFUA_3G00600*. The remaining two variants were found in putative oxidoreductases: a missense variant in *AFUA_3G00620*, encoding a putative zinc-containing alcohol dehydrogenase, and in *AFUA_7G01050*, encoding for a putative salicylate hydroxylase. These two enzymes are involved in the oxidation-reduction process, a process relevant to AMB resistance in *A. fumigatus*. For example, AMB exposure has been reported to induce the production and accumulation of intracellular reactive oxygen species (ROS) in *A. fumigatus*, thereby resulting in oxidative damage [51]. Alcohol dehydrogenases catalyze the interconversion between alcohols and aldehydes or ketones [52]. Alcohol fermentation is carried out by many microorganisms in hypoxic environments to allow for the regeneration of NAD+, ensuring an adequate supply for the continuation of glycolysis [53]. However, the increased production of intracellular ROS is also seen in *A. fumigatus* when exposed to oxygen-limiting environments, which then triggers the oxidative stress response [54]. In addition, alcohol dehydrogenase can influence hypoxic fungal growth in invasive aspergillosis infections [53]. Meanwhile, salicylate hydroxylase is a flavin-dependent monooxygenase that catalyzes the conversion of salicylate into catechol [55]. Overexpression of salicylate hydroxylase in *Aspergillus nidulans* was found to be associated with terbinafine resistance [56]. However, the enzyme has not been linked to AMB resistance until now. However, terbinafine also induces intracellular ROS accumulation in *A. fumigatus* [54]. Both terbinafine and AMB can cause significantly higher levels of mitochondrial lipid oxidation than in unstressed mycelia [54]. Therefore, in addition to naphthalene degradation, salicylate hydroxylase could potentially play a role in antifungal drug resistance through oxidative stress protection.

Surprisingly, we found no overlap in the top 20 SNPs identified based on GWAS between our previous study [36] and the current study. The difference in results is most likely attributed to factors such as changes in sample size, selection criteria, and analytical methods. Specifically, our previous GWAS focused on 33 Cluster II strains, while our current analysis included strains in all three clades. Additionally, the software used for the association analysis differed between our two studies, PLINK and TASSEL. Different GWAS software can produce dissimilar *p*-value ranking results, even when using the same input file. This was seen in a recent *A. fumigatus* study that compared overlapping SNPs between software TASSEL and RoadTrips [57]. Here, we conducted an AMB GWAS comparison examining TASSEL and PLINK results using the previous study’s dataset of the 33 Clade II *A. fumigatus* strains, with quality control threshold criteria of an MAF of 0.05, a quality score of 20, the removal of indels and excluding genotypes called below 95% across all individuals. Our results revealed that with a LOD score > 2.4, 36 and 57 SNPs were found by TASSEL and PLINK, respectively. Among these SNPs, 18 were found overlapping between the two softwares. However, a greater overlap in the number of significant SNPs was found between these two approaches for triazole resistance in *A. fumigatus* [37]. Specifically, using an LOD score threshold of 3 and filtration settings of “QUAL > 20, QUAL/AO > 10, SAF > 0, SAR > 0, RPR > 1, RPL > 1, DP > 10, MQM > 30 and MQMR > 30”, the itraconazole GWAS comparison showed 31 overlapping SNPs between these two approaches, with 7 unique SNPs found only by TASSEL. The voriconazole GWAS comparison found 44 overlapping SNPs, with 15 found only by TASSEL [37]. Since TASSEL produced a more conservative number of significant SNPs and likely fewer false positives, here, we focused on results from the TASSEL approach. However, confirmation of our resulting 20 SNPs putatively associated with AMB resistance via additional experiments (such as genetic crosses and gene replacements, similarly to those by Zhao et al. [57]) is still needed.

Linkage disequilibrium analysis, conducted on the top 20 SNPs and the 277,669 SNPs of the soft-filtered VCF file, identified an additional 24 highly linked (R^2^ > 0.85) variants among the 98 strains. Fisher’s exact tests identified 8 of the 24 SNPs to be significantly associated with AMB resistance (Table 3). Among these eight SNPs, five were intergenic variants and comprised of four SNPs in the intergenic region between *AFUA_4G09240* and *AFUA_4G09250*, which both encode for uncharacterized proteins, and one intergenic variant between *AFUA_5G00700* and *AFUA_5G00710*, encoding for an uncharacterized protein and a putative gamma-aminobutyric acid (GABA) permease, respectively. These intergenic variants could impact the gene expressions of the surrounding genes and targeted RT-qPCR analyses could help confirm their effects [58]. Two of the eight significantly associated SNPs were missense variants. One missense variant was in *AFUA_5G00710*, which encoded for a putative GABA permease, and the other was found in *AFUA_5G09220*, encoding a BEACH (Beige and Chediak-Higashi) domain protein. The final SNP was a non-coding transcript variant in *AFUA_5G09320*, which encodes for a putative signal transduction protein (Syg1) with plasma membrane localization. The non-coding mutation can also impact gene expression or function if located in elements such as enhancers, silencers, promoters or other regulatory roles.

Fisher’s exact tests were also performed on the 12 missense variants that were found in our previous study to be significantly associated with AMB resistance using a significance threshold *p*-value of 0.05 [36]. Among these 12 SNPs, 6 were found to be significantly associated with AMB resistance using our current 98-strain sample set and a Bonferroni-corrected *p*-value threshold of 1.39 × 10^−3^ (0.05/36). These six missense variants were in the three genes *tcsB* (*n* = 3), *mpkC* (*n* = 2), and *catA* (*n* = 1). The Bonferroni-corrected *p*-value was used here to reduce the number of false-positive SNPs. However, if the *p*-value threshold of 0.05 was used in the current study, four additional SNPs, including those of *erg3*, would remain significantly associated with AMB resistance (Table 4). As mentioned in the previous study, the genes *tcsB* and *mpkC* are involved in the high-osmolarity glycerol (HOG) mitogen-activated protein kinase (MAPK) signaling pathway, encoding for a sensor histidine kinase and mitogen-activated protein kinase, respectively [36]. The third gene, *catA*, which encodes for a catalase, was also included due to its role in the ROS-detoxifying system [36]. Missense variants in these genes were examined because of their involvement in oxidative stress response pathways, and thus their potential involvement in AMB resistance through protection against oxidative stress. However, the molecular roles of these specific genes in AMB susceptibility remain unknown.

In this study, a genetic cross was conducted between CM11 and AFB62-1 to generate 143 progeny strains. The result of this cross showed a wide variation in progeny growth values in varying AMB concentrations, with many progeny strains exhibiting transgressive phenotypes, with growths being either two standard deviations higher than the fast-growing parental strain or two standard deviations lower than the slow-growing parental strain. The results here are consistent with multiple quantitative trait loci influencing the growths of *A. fumigatus* in varying AMB concentrations. Specifically, our results suggest that parental strains CM11 and AFB62-1 differ at multiple loci that contribute to fungal growth differences in varying AMB concentrations, with strain CM11 having advantageous alleles (those contributing to greater growth) at some loci and strain AFB62-1 possessing advantageous alleles at other loci. Since *A. fumigatus* is a haploid, mating between these two parental strains followed by sexual recombination would generate some progeny with more or fewer advantageous allele combinations than either parental strain. Here, we focused on experimentally investigating the effects of five of the top SNPs identified above on AMB susceptibility and fungal growths under various AMB concentrations using the cross between CM11 and AFB62-1. As described in Section 2.3 “Materials and Methods”, these five significant SNPs had different genotypes between two AMB resistant strains in our collection that also had different mating types. The parental strains were chosen to test whether these five SNPs were associated with MIC differences and/or growth differences among progeny at different AMB concentrations. Interestingly, using the 143 progeny strains, the Fisher’s exact tests found no statistically significant differences between the MIC groups in their parental allele distributions (Table 6 and Table 7), consistent with no contribution to the MIC value differences (4 mg/L vs. 8 mg/L) between the two chosen parental strains. However, a new MIC class (2 mg/L) was found in the progeny, suggesting that the mechanisms of AMB resistance between the two parental strains were not identical. Furthermore, Welch’s t-tests revealed a significant difference in fungal growth at an AMB concentration of 0.25 mg/L between alleles at SNP site 5. In addition, we found significant interactions between SNP sites influencing progeny growths at various AMB concentrations. Specifically, 6 of the 10 SNP combinations showed significant interaction effects for growths in at least one of the AMB concentrations (Figure 4). In several instances, progeny with allele combinations from one parent showed more robust growth than those from a different parent. This can be seen in the combination of SNPs 5 and 1, where progeny that inherited the CM11 genotype at both SNP sites had a higher mean fungal growth ratio at 0.25 mg/L than progeny that inherited both genotypes from AFB61-2 (Figure 4A). In other SNP combinations, progeny with recombinant genotypes showed greater growth than those with parental genotypes (Figure 4B–D). Examples of this type include combinations of SNP 5 and 2, SNP 5 and 3, and SNP 5 and 4, where progeny that inherited the CM11 allele at SNP 5 and the AFB62-1 allele at the second SNP site (SNP 2, SNP 3, and SNP 4, respectively) had higher fungal growth rates than others (Figure 4B–D). This interaction pattern was also seen after combining SNP sites showing significant linkage disequilibrium (Figure 5A). Together, these results reveal that progeny growths in various AMB concentrations were influenced by different but sometimes overlapping SNP combinations. Interestingly, both parental and recombinant genotypes showed positive associations with growths at different AMB concentrations. Together, the results are consistent with the two parental strains being genetically very different, with complementary alleles at different SNP loci related to growths at different AMB concentrations.

Among these five SNP sites, SNP 1 was an intergenic variant between *AFUA_5G00700* and *AFUA_5G00710*, which encode for an uncharacterized protein and a putative GABA permease, respectively. GABA permeases serve as gamma-aminobutyrate transporter proteins and are involved in the utilization of GABA as a nitrogen and carbon source [59]. SNP 2 was an intergenic variant found between *AFUA_5G09190* and *AFUA_5G09200*. The gene *AFUA_5G09190* encodes a putative ABC bile acid transporter, part of the ABC transporter superfamily with many members involved in antifungal drug resistance, while *AFUA_5G09200* encodes a putative ubiquitin conjugating enzyme, UbcC. Ubiquitin conjugating enzymes are responsible for the ubiquitination or ubiquitin-like modification of proteins, which plays a role in many biological processes [60]. The next variant, SNP 3, was a missense mutation in *AFUA_5G09220*, a BEACH domain protein sequence. Their exact biological function remains largely unknown; however, BEACH domain proteins have been implicated in membrane dynamics, vesicular transport, and receptor signaling [61]. SNP 4 was a missense variant in *AFUA_5G09320*, encoding a putative signal transduction protein (Syg1) with plasma membrane localization. Although the protein’s function is not clear, Syg1 is predicted to be involved in phosphate homeostasis and to mediate phosphate export due to its similarity to the mammalian phosphate exporter Xpr1 [62]. The final variant site, SNP 5, was an intergenic variant located between *AFUA_6G07160* and *AFUA_6G07170*, encoding for a putative IZH family channel protein (Izh3) and an uncharacterized protein, respectively. The IZH family consists of membrane proteins involved in zinc homeostasis [63]. These genes are regulated by exogenous fatty acids, suggesting a role in lipid metabolism, and have been proposed to affect zinc homeostasis by altering sterol metabolism [63]. Interestingly, in a previous study on *Saccharomyces cerevisiae*, *izh3* deletion mutants were more resistant to AMB than the wild-type strain [64]. Furthermore, AMB had no significant effect on ROS production in the deletion mutants, but was significantly induced in the wild-type strain [64].

Our study here showed that genetic cross can be an effective approach for investigating the effects of candidate SNPs, as revealed by the GWAS on AMB susceptibility in *A. fumigatus*. However, we would like to note that the two parental strains used to construct our genetic cross were chosen for their specific traits, such as being different at several SNPs easily distinguished by PCR-RFLP, available in our strain collection, capable of efficient mating and sexual spore production, and both having high AMB MIC. While these features allowed us to identify several of the interesting phenomena reported here, including the multigenic nature of AMB susceptibility and the two parental strains having different AMB resistance mutations, other types of crosses such as those involving a high AMB MIC parent and a low AMB MIC parent may generate broader phenotypic categories among progenies, and enable the mapping and confirmation of additional SNPs associated with AMB resistance. 

At present, most studies of antifungal drug resistance classify strains into binary categories: resistant vs. susceptible. In this study, we have focused on the quantitative nature of both AMB MIC values and fungal growths at different AMB concentrations. As shown here, we believe analyzing quantitative variation is crucial for understanding the complexities of antifungal drug resistance. The MIC breakpoints used to define and separate *A. fumigatus* strains (as well as strains in other human fungal pathogens) into resistant and susceptible categories have changed over time for several antifungal drugs; therefore, rather than restricting our analysis to the predominant binary classification threshold, analyzing MIC differences enabled us to reveal the polygenic aspect of AMB susceptibility using the GWAS and laboratory cross. In addition, with quantitative fungal growths within hosts being an important clinical consideration, variations among strains in terms of their growth abilities, with and without antifungal drugs, should be more frequently analyzed. Our analyses of *A. fumigatus* growths here identified mutations and SNP–SNP interactions significantly associated with fungal growth inhibition at various AMB concentrations, which were not found to be significant when using MIC value differences.

In this research, our focus was on the relationship between genomic SNPs and AMB susceptibility in *A. fumigatus*. However, other genomic features not analyzed here, such as the copy number variation of genes and the presence of mycoviruses, may also influence AMB susceptibility (and other phenotypes) in this species. For example, the tandem duplication of a 34 bp or a 46 bp sequence in the promoter region of *cyp51a* is associated with an elevated MIC to triazole antifungals in *A. fumigatus* [37]. Copy number variations of genes were not analyzed for their influences on AMB susceptibility in our current study. In addition, several mycoviruses have been found in *Aspergillus* fungi, and some of them are known to impact phenotypes in these fungal species [65,66,67]. For example, Takahashi-Nakaguchi et al. [66] showed that the presence of a double-stranded RNA virus AfuCV41362 in *A. fumigatus* strain IFM 41362 had reduced tolerance to hypoxic, nitrosative, oxidative, and osmotic stresses. Furthermore, the virus-infected strain IFM 41362 had lower virulence than the virus-free strain of the same genotype in a mouse infection model [66]. While the influence of this and other potential mycoviruses to AMB susceptibility in *A. fumigatus* has not been systematically investigated, given their impacts on stress responses, it is possible that these viruses could contribute to AMB susceptibility differences in *A. fumigatus*. In our study, the distributions of mycoviruses among the genome-sequenced strains analyzed here are unknown, and thus their relationships to AMB susceptibility could not be analyzed. Because most known mycoviruses in *Aspergillus* fungi are RNA viruses [67], RNA-seq data (in addition to the genome-sequence data analyzed here) are needed to determine the distributions of these mycoviruses and their potential relationships to AMB susceptibility differences in *A. fumigatus*.

In recent years, advancements in medical technology and the increased usage of immunosuppressive agents have led to an expanding population of immunocompromised hosts, as well as a rising incidence of invasive mycoses such as aspergillosis. With the recommendation for a shift to AMB use in first-line invasive aspergillosis treatment where triazole resistance rates exceed 10%, the emerging problem of widespread AMB resistance and reports of high resistance rates—27% in Campinas, Brazil and 96.4% in Hamilton, Canada—are becoming a major public health concern [28,34,35]. This study has identified a total of 34 SNP candidates putatively associated with AMB susceptibility, and has highlighted the importance of SNP–SNP interactions in AMB susceptibility for 5 of these SNPs. The variants and genomic regions we have identified in this study provide promising candidates for future studies exploring molecular mechanisms for AMB susceptibility in *A. fumigatus*, and for further functional analysis. Furthermore, these candidates can help to accelerate the selection of prospective gene markers for AMB resistance screening in *A. fumigatus*. The development and clinical applications of molecular markers such as those identified here into rapid diagnostic kits could significantly shorten the time for drug-resistance identification, facilitate targeted treatment at early stages of infection, and reduce mobility and mortality caused by *A. fumigatus* and other fungal pathogens.

## Figures and Tables

**Table 1:** The primers, amplification conditions, and restriction enzymes used for distinguishing the five SNP sites between parental strains CM11 and AFB62-1 as well as their progeny.

SNP Site Number	Chromosome and Position (bp)	Primer Sequence (5′ to 3′)	Amplification Conditions	Restriction Enzyme
1	CHR 5—201,094	F: ACAAACGCCCTTGATCGCTA R: TTTGAGCAGGCCGTAGAGTG	95 °C for 10 min; 40 cycles: 95 °C for 30 s, 56 °C for 30 s, 72 °C for 1 min; 72 °C for 5 min.	*Fau*I
2	CHR 5—2,362,267 (Represented by CHR 5—2,362,923)	F: CCCTAATGGGTCCGCCAAAA R: CCAGGTGGGGAGTATGGGTA	95 °C for 10 min; 40 cycles: 95 °C for 30 s, 57 °C for 30 s, 72 °C for 1 min; 72 °C for 5 min.	*HpyCH*4IV
3	CHR 5—2,370,937	F: GCCTACAGGGTCTTGCTTGT R: TGTCAGGACCGCCAATGAAA	95 °C for 10 min; 40 cycles: 95 °C for 30 s, 56 °C for 30 s, 72 °C for 1 min; 72 °C for 5 min.	*Bbs*I
4	CHR 5—2,399,121	F: ATGAGGCAAGGGATCGTACC R: TGCCTACCTCAATCGCACTG	95 °C for 10 min; 40 cycles: 95 °C for 30 s, 56 °C for 30 s, 72 °C for 1 min; 72 °C for 5 min.	*HpyCH*4III
5	CHR 6—1,608,813 (Represented by CHR 6—1,608,090)	F: AAGACAACTTCCGAGCCGTG R: GCCCCTCTTGGCCTCATTT	95 °C for 10 min; 40 cycles: 95 °C for 30 s, 57 °C for 30 s, 72 °C for 1 min; 72 °C for 5 min.	*BspD*I

**Table 2:** The top 20 SNPs associated with AMB susceptibility, arranged based on −log_10_(*p*-Values).

Chromosome	Position (bp)	Change	−log_10_ (*p*-Value)	Gene ID	Annotation	Predicted Effect
7	278,099	A to G	4.49	*AFUA_7G01030-AFUA_7G01040*	Calcium-transporting ATPase—Cytidine deaminase, putative	Intergenic Region
5	2,362,267	G to A	3.46	*AFUA_5G09190-AFUA_5G09200*	ABC bile acid transporter, putative—Ubiquitin conjugating enzyme (UbcC), putative	Intergenic Region
5	2,386,509	T to G	3.38	*AFUA_5G09260-AFUA_5G09270*	Phosphatidylinositol transporter, putative—ER membrane protein complex subunit 1	Intergenic Region
4	3,275,045	T to A	3.36	*AFUA_4G12480*	Asparagine synthase-related protein	Missense Variant (Ser424Cys)
2	4,385,926	A to G	3.20	*AFUA_2G16500-AFUA_2G16510*	Uncharacterized protein—Uncharacterized protein	Intergenic Region
1	3,787,543	A to G	3.15	*AFUA_1G00400-AFUA_1G00420*	Uncharacterized protein—Carboxypeptidase	Intergenic Region
5	3,698,701	G to T	3.08	*AFUA_5G14160-AFUA_5G14170*	Uncharacterized protein—Uncharacterized protein	Intergenic Region
6	1,608,813	C to T	3.06	*AFUA_6G07160-AFUA_6G07170*	IZH family channel protein (Izh3), putative—Uncharacterized protein	Intergenic Region
3	1,260,557	T to C	2.99	*AFUA_3G04310-AFUA_3G05320*	SnoRNA-binding protein, putative—C2H2 finger domain protein, putative	Intergenic Region
6	3,521,360	G to A	2.85	*AFUA_6G13770-AFUA_6G13780*	C6 finger domain protein, putative—MFS multidrug transporter, putative	Intergenic Region
6	3,141,751	G to A	2.83	*AFUA_6G12420*	SprT family metallopeptidase, putative	Missense Variant (Glu245Lys)
6	3,149,653	G to T	2.83	*AFUA_6G12460*	Uncharacterized protein	Missense Variant (Asn213Lys)
3	133,642	T to C	2.82	*AFUA_3G00600*	Uncharacterized protein	Missense Variant (Val519Ala)
3	142,183	A to C	2.81	*AFUA_3G00620*	Zinc-containing alcohol dehydrogenase, putative	Missense Variant (His136Pro)
7	1,182,007	A to C	2.81	*AFUA_7G05020-AFUA_7G05030*	Polysaccharide export protein (Cap59), putative—Pectin lyase B	Intergenic Region
7	279,416	T to C	2.80	*AFUA_7G01050*	Salicylate hydroxylase, putative	Missense Variant (Gln396Arg)
4	2,417,511	A to G	2.75	*AFUA_4G09240-AFUA_4G09250*	Uncharacterized protein—Uncharacterized protein	Intergenic Region
4	2,417,525	T to G	2.75	*AFUA_4G09240-AFUA_4G09250*	Uncharacterized protein—Uncharacterized protein	Intergenic Region
3	3,512,400	T to C	2.71	*AFUA_3G13230*	AT DNA-binding protein, putative	Synonymous Variant (Pro380Pro)
3	3,122,663	A to C	2.70	*AFUA_3G11850-AFUA_3G11860*	Uncharacterized protein—Microtubule associated protein EB1, putative	Intergenic Region

**Table 3:** Additional variants found through linkage disequilibrium analysis to be highly linked with the top 20 SNPs from the AMB GWAS. Fisher’s exact test *p*-values, comparing AMB-resistant and susceptible strains, are listed (*n* = 98).

Chromosome	Position	Gene ID	Predicted Effect (Amino Acid Substitution)	Description	Fisher’s Exact Tests (*p*-Value)
1	3,782,532	*AFUA_1G14160*	Missense Variant (Ser65Phe)	Uncharacterized protein	1.96 × 10^−1^
1	3,787,813	*AFUA_1G00400-AFUA_1G00420*	Intergenic Region	Uncharacterized protein—Carboxypeptidase	3.42 × 10^−1^
1	3,796,235	*AFUA_1G00400-AFUA_1G00420*	Intergenic Region	Uncharacterized protein—Carboxypeptidase	3.43 × 10^−1^
1	3,800,222	*AFUA_1G00400-AFUA_1G00420*	Intergenic Region	Uncharacterized protein—Carboxypeptidase	1.90 × 10^−1^
1	3,801,124	*AFUA_1G00400-AFUA_1G00420*	Intergenic Region	Uncharacterized protein—Carboxypeptidase	1.96 × 10^−1^
1	3,801,488	*AFUA_1G00400-AFUA_1G00420*	Intergenic Region	Uncharacterized protein—Carboxypeptidase	1.96 × 10^−1^
1	3,801,524	*AFUA_1G00400-AFUA_1G00420*	Intergenic Region	Uncharacterized protein—Carboxypeptidase	1.96 × 10^−1^
1	3,801,974	*AFUA_1G00400-AFUA_1G00420*	Intergenic Region	Uncharacterized protein—Carboxypeptidase	1.96 × 10^−1^
1	3,802,717	*AFUA_1G00400-AFUA_1G00420*	Intergenic Region	Uncharacterized protein—Carboxypeptidase	1.88 × 10^−1^
1	3,803,746	*AFUA_1G14240*	Missense Variant (Glu467Asp)	Uncharacterized protein	1.99 × 10^−1^
3	142,511	*AFUA_3G00620*	Synonymous Variant (Val245Val)	Zinc-containing alcohol dehydrogenase, putative	6.67 × 10^−1^
3	3,129,756	*AFUA_3G11890*	Non-coding Transcript Variant	Thermolabile L-asparaginase, putative	1.06 × 10^−1^
4	2,416,428	*AFUA_4G09240-AFUA_4G09250*	Intergenic Region	Uncharacterized protein—Uncharacterized protein	3.39 × 10^−7^ *
4	2,417,416	*AFUA_4G09240-AFUA_4G09250*	Intergenic Region	Uncharacterized protein—Uncharacterized protein	1.28 × 10^−6^ *
4	2,417,517	*AFUA_4G09240-AFUA_4G09250*	Intergenic Region	Uncharacterized protein—Uncharacterized protein	2.96 × 10^−4^ *
4	2,417,806	*AFUA_4G09240-AFUA_4G09250*	Intergenic Region	Uncharacterized protein—Uncharacterized protein	2.58 × 10^−4^ *
5	201,094	*AFUA_5G00700-AFUA_5G00710*	Intergenic Region	Uncharacterized protein—GABA permease, putative	7.12 × 10^−4^ *
5	201,751	*AFUA_5G00710*	Missense Variant (Arg37Lys)	GABA permease, putative	7.12 × 10^−4^ *
5	2,370,937	*AFUA_5G09220*	Missense Variant (Leu872Val)	BEACH domain protein	5.15 × 10^−4^ *
5	2,399,121	*AFUA_5G09320*	Non-coding Transcript Variant	Signal transduction protein (Syg1), putative	7.64 × 10^−4^ *
6	3,132,855	*AFUA_6G12400-AFUA_6G12410*	Intergenic Region	1,3-beta-D-glucan-UDP glucosyltransferase—1,3-beta-glucanosyltransferase	7.28 × 10^−1^
6	3,136,524	*AFUA_6G12400-AFUA_6G12410*	Intergenic Region	1,3-beta-D-glucan-UDP glucosyltransferase—1,3-beta-glucanosyltransferase	7.27 × 10^−1^
6	3,148,083	*AFUA_6G12440-AFUA_6G12450*	Intergenic Region	Uncharacterized protein—Chaperone/heat shock protein (Hsp12), putative	7.40 × 10^−1^
7	1,184,553	*AFUA_7G05030-AFUA_7G05040*	Intergenic Region	Pectin lyase B—Rhamnosidase B, putative	3.18 × 10^−1^

* Statistically significant SNPs based on Bonferroni-corrected significance threshold of *p* < 1.39 × 10^−3^.

**Table 4:** Fisher’s exact tests comparing AMB resistant and susceptible strains on the 12 previously found missense variants associated with AMB resistance (*n* = 98).

Chromosome	Position (bp)	Gene	Amino Acid Substitution	Fisher’s Exact Test (*p*-Value)
2	61,543	*AFUA_2G00320* (*erg3*)	Thr154Ile	3.75 × 10^−2^
2	62,002	*AFUA_2G00320* (*erg3*)	Tyr286Phe	3.75 × 10^−2^
2	145,934	*AFUA_2G00660* (*tcsB*)	Asp759Gly	6.10 × 10^−4^ *
2	146,469	*AFUA_2G00660* (*tcsB*)	Gly581Ser	4.27 × 10^−3^
2	147,363	*AFUA_2G00660* (*tcsB*)	Arg283Gly	1.32 × 10^−3^ *
2	147,396	*AFUA_2G00660* (*tcsB*)	Ala272Pro	4.39 × 10^−4^ *
5	2,342,264	*AFUA_5G09100* (*mpkC*)	Trp330Ser	4.43 × 10^−5^ *
5	2,342,466	*AFUA_5G09100* (*mpkC*)	Ile378Thr	4.43 × 10^−5^ *
6	857,963	*AFUA_6G03890* (*catA*)	Asp328Asn	5.28 × 10^−2^
6	858,366	*AFUA_6G03890* (*catA*)	Ser462Asn	1.48 × 10^−4^ *
6	2,533,399	*AFUA_6G10240* (*fos1*)	Ala532Asp	8.17 × 10^−2^
6	3,232,955	*AFUA_6G12820* (*mpkB*)	Lys272Arg	3.23 × 10^−2^

* Statistically significant SNPs based on Bonferroni-corrected significance threshold of *p* < 1.39 × 10^−3^.

**Table 5:** Information about the five SNP sites that were genotyped in the progeny strains using PCR-RFLP.

SNP ID	Chromosome	Position (bp)	Gene ID	Annotation	Predicted Effect
1	5	201,094	*AFUA_5G00700-AFUA_5G00710*	Uncharacterized protein—GABA permease, putative	Intergenic Region
2	5	2,362,267	*AFUA_5G09190-AFUA_5G09200*	ABC bile acid transporter, putative—Ubiquitin conjugating enzyme (UbcC), putative	Intergenic Region
3	5	2,370,937	*AFUA_5G09220*	BEACH domain protein	Missense Variant (Leu872Val)
4	5	2,399,121	*AFUA_5G09320*	Signal transduction protein (Syg1), putative	Non-coding Transcript Variant
5	6	1,608,813	*AFUA_6G07160-AFUA_6G07170*	IZH family channel protein (Izh3), putative—Uncharacterized protein	Intergenic Region

**Table 6:** Allele distribution at five SNP sites among the 143 progeny strains. The variant alleles are separated based on AMB MIC groups (MIC = 2 mg/L, 4 mg/L or 8 mg/L). Fisher’s exact tests (3 × 2 contingency table) were conducted between MIC groups and the inherited parental allele, with *p*-values listed. Differences were considered statistically significant at *p* < 0.01.

	MIC = 2 mg/L		MIC = 4 mg/L		MIC = 8 mg/L		Fisher’s Exact Test (*p*-Value)
	Allele 1	Allele 2	Allele 1	Allele 2	Allele 1	Allele 2	
SNP 1	0	4	64	56	7	12	4.89 × 10^−2^
SNP 2	1	3	59	61	7	12	4.00 × 10^−1^
SNP 3	1	3	60	60	8	11	6.00 × 10^−1^
SNP 4	1	3	63	57	9	10	6.00 × 10^−1^
SNP 5	3	1	53	67	8	11	5.42 × 10^−1^

Allele 1 = AFB62-1, Allele 2 = CM11.

**Table 7:** Distribution of pairwise genotype combinations at five SNP sites among the 143 progeny strains. The variant alleles are separated based on AMB MIC groups (MIC = 2 mg/L, 4 mg/L or 8 mg/L). Fisher’s exact tests (4 × 3 contingency table) were conducted between MIC groups and the inherited parental allele, with *p*-values listed. Differences were considered statistically significant at *p* < 0.005.

	MIC = 2 mg/L				MIC = 4 mg/L				MIC = 8 mg/L				Fisher’s Exact Test (*p*-Values)
	Alleles 1 and 1	Alleles 1 and 2	Alleles 2 and 1	Alleles 2 and 2	Alleles 1 and 1	Alleles 1 and 2	Alleles 2 and 1	Alleles 2 and 2	Alleles 1 and 1	Alleles 1 and 2	Alleles 2 and 1	Alleles 2 and 2	
SNP 5 and 4	1	2	0	1	31	22	32	35	5	3	4	7	7.99 × 10^−1^
SNP 5 and 3	1	2	0	1	29	24	31	36	4	4	4	7	8.64 × 10^−1^
SNP 5 and 2	1	2	0	1	28	25	31	36	4	4	3	8	7.27 × 10^−1^
SNP 5 and 1	0	3	0	1	29	24	35	32	2	6	5	6	2.15 × 10^−1^
SNP 4 and 3	1	0	0	3	58	5	2	55	8	1	0	10	7.76 × 10^−1^
SNP 4 and 2	1	0	0	3	56	7	3	54	7	2	0	10	7.57 × 10^−1^
SNP 4 and 1	0	1	0	3	33	30	31	26	3	6	4	6	3.03 × 10^−1^
SNP 3 and 2	1	0	0	3	58	2	1	59	7	1	0	11	5.07 × 10^−1^
SNP 3 and 1	0	1	0	3	34	26	30	30	3	5	4	7	3.56 × 10^−1^
SNP 2 and 1	0	1	0	3	33	26	31	30	3	4	4	8	3.07 × 10^−1^

## Data Availability

Accession numbers for all 98 whole-genome sequences used in this study can be found listed in Supplementary Table S1.

## Supplementary Materials

The following are available online at https://www.mdpi.com/article/10.3390/jof7100860/s1, Figure S1: Quantile–Quantile (QQ) plot of the Amphotericin B GWAS, Figure S2: Growths of the 143 progeny strains in varying Amphotericin B concentrations, grouped based on the variant genotype at the sites (A) SNP 1, (B) SNP 2, (C) SNP 3, (D) SNP 4, and (E) SNP 5. Fungal growth was determined by calculating difference in OD_530_ at start (0 h) and end of incubation (48 h); this value was divided by fungal growth in the positive control to determine ratio of fungal growth. Welch’s *t*-test *p*-values are listed to compare variant genotype. Parental allele 1 denotes the AFB62-1 genotype and parental allele 2 denotes the CM11 genotype. “‡” indicates bar groups with *n* ≤ 12, Figure S3: Welch’s t-test *p*-values for the 10 pairwise SNP combinations in their associations with fungal growths at different Amphotericin B concentrations, Table S1: Information on the 98 *A. fumigatus* strains analyzed in this study, Table S2: Amphotericin B susceptibility and genotype information for the two parental and their 143 progeny strains, Table S3: Parental genotype at the 28 SNP sites of interest associated with Amphotericin B susceptibility.

## Appendix A

Common Acronyms and Abbreviations:

AMB	Amphotericin B
GWAS	Genome-wide association study
MIC	Minimum inhibitory concentration
PCR	Polymerase chain reaction
RFLP	Restriction fragment length polymorphism
ROS	Reactive oxygen species
SNP	Single-nucleotide polymorphism
