# Supplementary material for: Genetic Analyses of Amphotericin B Susceptibility in Aspergillus fumigatus
Source: J Fungi (Basel). 2021 Oct 14;7(10):860. doi: 10.3390/jof7100860 (PMC8538161; doi:10.3390/jof7100860)
Supplement: Supplementary file 1 [file jof-07-00860-s001.zip › Supplementary Table S1.pdf]

Strain name	Run	Accession	Project Number	Country	Source	AMB MIC (mg/L)	Reference DOI
Afs35	DRR146814	DRS075098	PRJDB7240	Germany	Clinical	1	10.1371/journal.ppat.1006096
B5233	ERR2863830	ERS2867139	PRJEB28819	United States	Clinical	0.5	10.3201/eid1508.090251
Af65	ERR769499	ERS663171	PRJEB8623	United Kingdom	Clinical	0.5	10.1128/mBio.00536-15
12-7505446	ERR769500	ERS663164		United Kingdom	Clinical	0.25	
12-7505220	ERR769501	ERS663165		United Kingdom	Clinical	0.5	
09-7500806	ERR769502	ERS663166		United Kingdom	Clinical	0.5	
12-7504652	ERR769503	ERS663167		United Kingdom	Clinical	0.25	
12-7504462	ERR769504	ERS663168		United Kingdom	Clinical	0.5	
12-7505054	ERR769505	ERS663169		United Kingdom	Clinical	0.5	
08-12-12-13	ERR769506	ERS663172		Netherlands	Clinical	0.25	
08-36-03-25	ERR769507	ERS663173		Netherlands	Clinical	0.5	
08-31-08-91	ERR769508	ERS663174		Netherlands	Clinical	0.5	
08-19-02-61	ERR769509	ERS663175		Netherlands	Environmental	0.25	
08-19-02-30	ERR769510	ERS663176		Netherlands	Environmental	0.5	
10-01-02-27	ERR769511	ERS663177		Netherlands	Clinical	0.5	
08-19-02-46	ERR769512	ERS663178		Netherlands	Environmental	0.5	
08-19-02-10	ERR769513	ERS663179		Netherlands	Environmental	0.25	
Afu 942/09	ERR769514	ERS663180		India	Clinical	0.25	
Afu 1042/09	ERR769515	ERS663181		India	Clinical	0.25	
Afu 343/P/11	ERR769516	ERS663182		India	Clinical	0.125	
Afu 591/12	ERR769517	ERS663183		India	Clinical	0.5	
Afu 124/E11	ERR769518	ERS663184		India	Environmental	0.125	
Afu 257/E11	ERR769520	ERS663186		India	Environmental	0.125	
Afu 218/E11	ERR769521	ERS663187		India	Environmental	0.125	
CM3262	SRR7418922	SRS3453382	PRJNA477519	Spain	Clinical	0.125 to 0.25 *	10.1128/AAC.00241-18
CM2733	SRR7418923	SRS3453380		Spain	Clinical	0.125 to 0.25 *	
CM2730	SRR7418924	SRS3453381		Spain	Clinical	0.125 to 0.25 *	
CM7560	SRR7418925	SRS3453379		Spain	Clinical	0.125 to 0.25 *	
CM3249	SRR7418926	SRS3453378		Spain	Clinical	0.125 to 0.25 *	
CM4602	SRR7418928	SRS3453376		Spain	Clinical	0.125 to 0.25 *	
CM2495	SRR7418930	SRS3453373		Spain	Clinical	0.125 to 0.25 *	
akuBKU80	SRR7418932	SRS3453372		Spain	Clinical	0.125 to 0.25 *	
CM7570	SRR7418933	SRS3453371		Spain	Clinical	0.125 to 0.25 *	
TP12	SRR7418940	SRS3453364		Spain	Clinical	0.125 to 0.25 *	
CM7632	SRR7418941	SRS3453363		Spain	Clinical	0.125 to 0.25 *	
TP32	SRR7418946	SRS3453358		Spain	Clinical	0.125 to 0.25 *	

CM4946	SRR7418948	SRS3453357		Spain	Clinical	0.125 to 0.25 *	
IFM 59355-1	DRR022916	DRS016460	PRJDB3064	Japan	Clinical	2	10.1128/JCM.01105-14
IFM 59355-2	DRR022917	DRS016461		Japan	Clinical	2	
IFM 59356-1	DRR022919	DRS016462		Japan	Clinical	2	
IFM 59356-2	DRR022922	DRS016463		Japan	Clinical	1	
IFM 59356-3	DRR022924	DRS016464		Japan	Clinical	2	
IFM 59361-1	DRR022925	DRS016465		Japan	Clinical	1	
IFM 59361-2	DRR022926	DRS016466		Japan	Clinical	1	
IFM 60237	DRR022927	DRS016467		Japan	Clinical	2	
IFM 55369	DRR015087	DRS013318	PRJDB1541	Japan	Clinical	1	10.1016/j.jiac.2015.01.005
IFM 59056	DRR015093	DRS013319		Japan	Clinical	2	
IFM 59359	DRR015096	DRS013320		Japan	Clinical	2	
IFM 59361	DRR015099	DRS013321		Japan	Clinical	2	
IFM 59073	DRR015106	DRS013323		Japan	Clinical	2	
IFM 58029	DRR017540	DRS015320		Japan	Clinical	0.5	
IFM 58401	DRR017543	DRS015321		Japan	Clinical	1	
IFM 59777	DRR017549	DRS015323		Japan	Clinical	0.5	
CNM-CM8689	SRR10592629	SRS5766627	PRJNA592352	Spain	Clinical	1	10.3389/fgene.2020.00459
CNM-CM8686	SRR10592630	SRS5766626		Spain	Clinical	0.5	
CNM-CM8812	SRR10592631	SRS5766625		Spain	Clinical	0.25	
CNM-CM8714	SRR10592632	SRS5766624		Spain	Clinical	0.25	
CNM-CM8057	SRR10592633	SRS5766623		Spain	Clinical	0.25	
DI 15-105	SRR7841980	SRS3786101	PRJNA491253	United States	Clinical	0.125	10.1128/mBio.00437-19
F11628	SRR343149	SRS260927	PRJNA67101	United Kingdom	Clinical	0.06	
F12865	SRR343150	SRS260928		United Kingdom	Clinical	0.125	
F12041	SRR617723	SRS375794		United Kingdom	Clinical	0.25	
F13535	SRR617726	SRS375797		United Kingdom	Clinical	0.125	
AF72 (NCPF 7099)	SRR617721	SRS375792		United States	Clinical	0.25 (by M38-A)	10.1093/jac/dkg384
AF90 (NCPF 7100)	SRR617722	SRS375793		United States	Clinical	0.5 (by M38-A)	
V130-15	SRR8759700	SRS4515816	PRJNA528395	Netherlands	Clinical	0.5	10.1016/j.fgb.2019.05.005
V157-62	SRR8759706	SRS4515810		Netherlands	Clinical	0.25	
CON4	SRR12894204	SRS7582162	PRJNA671765	Canada	Environmental	1	10.3390/microorganisms8111673
CM21	SRR12894203	SRS7582164		Canada	Environmental	2	
CM65	SRR12894200	SRS7582166		Canada	Environmental	4	
P80	SRR12894199	SRS7582167		Canada	Environmental	4	
15-1	SRR12894198	SRS7582170		Canada	Clinical	4	
AV88	SRR12894197	SRS7582168		Canada	Environmental	4	
15-33	SRR12894196	SRS7582169		Canada	Clinical	4	
P20	SRR12894195	SRS7582171		Canada	Environmental	4	

CM38	SRR12894194	SRS7582173		Canada	Environmental	4	
CM11	SRR12894193	SRS7582172		Canada	Environmental	8	
AFB62-1	SRR12894202	SRS7582163		United States	Clinical	4	
AFIR928	SRR12894201	SRS7582165		Ireland	Environmental	4	
IFM 46074	SRR11977812	SRS6816741	PRJNA638646	Japan	Clinical	0.5	10.1016/j.jiac.2015.01.005
IFM 48051	SRR11977855	SRS6816698		Japan	Clinical	1	
IFM 49896	SRR11977853	SRS6816700		Japan	Clinical	1	
IFM 50230	SRR11977852	SRS6816701		Japan	Clinical	1	
IFM 50916	SRR11977849	SRS6816704		Japan	Clinical	1	
IFM 50997	SRR11977847	SRS6816706		Japan	Clinical	1	
IFM 50999	SRR11977846	SRS6816707		Japan	Clinical	2	
IFM 51357	SRR11977844	SRS6816709		Japan	Clinical	2	
IFM 51505	SRR11977842	SRS6816711		Japan	Clinical	0.5	
IFM 51746	SRR11977841	SRS6816712		Japan	Clinical	2	
IFM 51977	SRR11977839	SRS6816714		Japan	Clinical	1	
IFM 51978	SRR11977838	SRS6816715		Japan	Clinical	0.5	
IFM 55369	SRR11977835	SRS6816718		Japan	Clinical	1	
IFM 59073	SRR11977828	SRS6816725		Japan	Clinical	2	
IFM 59359	SRR11977826	SRS6816727		Japan	Clinical	2	
CAPA-A	SRR12949929	SRS7620879	PRJNA673120	Germany	Clinical	2 to 4 *	10.1128/Spectrum.00010-21
CAPA-B	SRR12949928	SRS7620880		Germany	Clinical	2	
CAPA-C	SRR12949927	SRS7620882		Germany	Clinical	2	
CAPA-D	SRR12949926	SRS7620881		Germany	Clinical	2	

* The lower numeric value of a reported MIC range was used in the GWAS
