# Supplementary material for: Genetic Analyses of Amphotericin B Susceptibility in Aspergillus fumigatus
Source: J Fungi (Basel). 2021 Oct 14;7(10):860. doi: 10.3390/jof7100860 (PMC8538161; doi:10.3390/jof7100860)
Supplement: Supplementary file 1 [file jof-07-00860-s001.zip › Supplementary Table S2.pdf]

Strain ID	AMB MIC (mg/L)	Mean Ratio of Fungal Growth in varying AMB Concentrations					Genotype at five SNP sites (AFB62-1 = 1, CM11 = 2)				
		0.25 mg/L	0.50 mg/L	1.00 mg/L	2.00 mg/L	4.00 mg/L	SNP 1	SNP 2	SNP 3	SNP 4	SNP 5
AFB62-1	4	0.651	0.529	0.444	0.296	NA	1	1	1	1	1
CM11	8	0.773	0.672	0.418	0.338	0.006	2	2	2	2	2
1	4	0.752	0.605	0.494	0.007	NA	1	2	2	2	2
2	4	0.975	1.296	0.585	0.216	NA	1	1	1	1	1
3	4	0.799	0.681	0.668	0.187	NA	2	1	1	1	1
4	4	0.597	0.575	0.549	0.300	NA	2	2	2	2	1
5	4	0.591	0.439	0.383	0.127	NA	2	2	1	1	2
6	4	0.612	0.646	0.613	0.086	NA	1	1	1	1	1
7	8	0.938	0.772	0.578	0.523	0.193	2	2	1	1	2
8	4	0.893	0.770	0.714	0.559	NA	2	1	1	1	1
9	4	0.956	0.791	0.825	0.588	NA	1	1	1	1	2
10	4	0.777	0.705	0.575	0.212	NA	1	2	2	2	2
11	4	0.836	0.714	0.637	0.192	NA	1	1	1	1	1
12	4	0.835	0.586	0.584	0.106	NA	2	2	2	2	1
13	4	0.781	0.501	0.389	0.485	NA	1	1	1	1	2
14	4	1.059	0.943	0.878	0.507	NA	1	1	1	1	1
15	2	0.712	0.606	0.369	NA	NA	2	1	1	1	1
16	4	0.719	0.617	0.561	0.119	NA	2	2	2	2	2
17	8	1.140	1.049	1.206	0.684	0.020	2	2	2	2	1
18	4	0.859	0.531	0.422	0.188	NA	2	1	1	1	2
19	4	0.916	0.807	0.780	0.432	NA	1	1	1	1	1
20	4	0.779	0.800	0.827	0.583	NA	1	2	2	2	2
21	4	0.874	0.742	0.821	0.699	NA	2	1	1	1	2
22	4	0.821	0.602	0.559	0.362	NA	2	2	2	2	1
23	4	0.849	0.500	0.441	0.286	NA	2	1	1	1	2
24	4	1.327	0.836	1.024	0.295	NA	2	1	1	1	1
25	4	0.847	0.912	1.126	0.148	NA	1	2	2	2	2
26	4	0.782	0.811	1.227	0.085	NA	2	1	1	1	2
27	4	0.828	0.598	0.905	0.011	NA	1	1	1	1	1
28	4	1.381	1.113	0.956	0.689	NA	2	2	2	1	2
29	4	0.780	0.712	1.004	0.166	NA	1	1	1	1	2
30	2	0.783	0.706	0.504	NA	NA	2	2	2	2	2
31	2	0.626	0.426	0.304	NA	NA	2	2	2	2	1
32	4	0.898	0.766	1.068	0.414	NA	1	1	1	1	2
33	4	0.616	0.386	0.378	0.193	NA	2	1	1	1	1
34	4	0.547	0.310	0.205	0.053	NA	2	2	2	2	2
35	4	0.755	0.666	0.483	0.124	NA	2	2	2	2	2
36	4	0.660	0.730	0.832	0.530	NA	2	1	1	1	2
37	4	0.977	0.826	0.740	0.557	NA	1	2	2	2	1
38	4	0.795	0.400	0.383	0.250	NA	2	2	2	2	2
39	4	0.833	0.810	0.577	0.087	NA	2	2	2	2	2

40	4	1.006	0.731	0.637	0.427	NA	1	2	2	2	1
41	4	0.868	0.797	0.676	0.252	NA	1	1	1	1	2
42	4	0.788	0.612	0.484	0.357	NA	2	1	1	1	1
43	4	0.750	0.663	0.577	0.319	NA	1	1	1	1	1
44	8	0.896	0.810	0.809	0.733	0.008	2	2	2	2	2
45	4	0.678	0.417	0.277	0.179	NA	2	2	2	2	1
46	4	0.712	0.636	0.505	0.286	NA	2	2	2	2	1
47	4	0.838	0.778	0.625	0.292	NA	2	2	2	2	1
48	4	0.718	0.537	0.423	0.415	NA	1	2	2	2	2
49	4	0.652	0.394	0.278	0.170	NA	1	2	2	2	2
50	4	0.698	0.461	0.401	0.077	NA	1	2	2	1	1
51	8	0.864	0.842	0.783	0.645	0.247	2	1	1	1	1
52	8	0.682	0.715	0.586	0.616	0.088	2	1	1	1	1
53	8	0.445	0.484	0.398	0.459	0.040	1	2	2	2	2
54	4	0.741	0.487	0.484	0.238	NA	2	2	2	2	1
55	8	0.831	0.837	0.753	0.566	0.005	1	2	2	2	2
56	4	0.736	0.744	0.746	0.359	NA	1	1	1	1	2
57	4	0.910	0.758	0.444	0.276	NA	2	1	1	1	2
58	4	1.077	0.719	0.767	0.128	NA	2	2	2	2	1
59	4	0.851	0.592	0.573	0.495	NA	2	1	1	1	2
60	4	1.046	0.866	0.470	0.229	NA	1	1	1	1	1
61	8	0.923	0.903	0.855	0.733	0.074	2	2	2	1	1
62	4	0.692	0.703	0.682	0.469	NA	1	2	2	2	2
63	4	0.906	0.488	0.598	0.476	NA	1	2	2	2	1
64	4	0.739	0.594	0.460	0.287	NA	2	1	1	1	1
65	8	1.566	1.091	0.722	0.672	0.099	2	1	1	1	2
66	4	1.558	1.540	1.218	0.528	NA	2	1	1	1	2
67	4	0.844	0.861	0.837	0.653	NA	2	1	1	1	2
68	4	0.789	0.746	0.748	0.481	NA	2	2	2	1	2
69	4	0.955	1.408	0.633	0.077	NA	2	2	2	2	1
70	8	0.618	0.690	0.682	0.419	0.004	2	2	2	2	2
71	4	0.888	0.899	0.907	0.578	NA	1	2	2	2	2
72	4	1.759	0.762	0.822	0.240	NA	2	2	2	2	2
73	4	1.171	1.245	0.758	0.072	NA	1	1	1	1	2
74	4	0.655	0.735	0.724	0.283	NA	1	2	2	2	2
75	4	0.749	0.794	0.707	0.572	NA	1	1	1	1	2
76	4	0.928	0.832	0.814	0.549	NA	2	2	2	2	2
77	4	0.612	0.698	0.567	0.017	NA	2	1	1	1	2
78	8	0.594	0.645	0.740	0.307	0.017	1	1	1	1	1
79	4	0.771	0.802	0.474	0.131	NA	1	2	2	2	2
80	2	0.638	0.570	0.387	NA	NA	2	2	2	2	1
81	4	0.690	0.585	0.486	0.455	NA	1	1	1	1	2
82	4	0.877	1.020	0.691	0.389	NA	2	2	2	2	2

83	4	1.179	0.653	0.804	0.384	NA	1	2	2	2	2
84	4	0.765	0.517	0.549	0.317	NA	1	1	1	1	1
85	4	0.753	0.675	0.613	0.198	NA	2	2	2	2	2
86	4	0.672	0.549	0.416	0.156	NA	2	2	2	1	1
87	4	0.737	0.668	0.848	0.330	NA	1	1	1	1	1
88	4	0.833	0.796	0.482	0.291	NA	1	1	1	1	1
89	4	0.819	0.618	0.508	0.375	NA	2	2	2	2	2
90	4	0.697	0.736	0.762	0.305	NA	1	2	2	2	1
91	4	0.816	0.708	0.575	0.252	NA	1	2	2	2	2
92	4	0.563	0.526	0.442	0.171	NA	1	2	2	2	2
93	8	0.847	0.742	0.714	0.378	0.241	1	2	2	2	2
94	8	0.822	0.779	0.446	0.492	0.081	1	1	1	1	1
95	8	0.695	0.486	0.503	0.265	0.066	1	1	1	1	2
96	4	0.862	0.774	0.645	0.250	NA	2	1	1	1	2
97	4	0.808	0.659	0.637	0.166	NA	1	2	2	2	1
98	4	0.896	0.827	0.596	0.496	NA	2	1	1	1	2
99	4	0.695	0.863	0.704	0.125	NA	1	2	2	2	1
100	4	0.718	0.683	0.700	0.494	NA	1	2	2	2	1
101	4	0.790	0.637	0.588	0.231	NA	2	1	1	1	1
102	4	0.774	0.974	0.803	0.562	NA	1	2	2	2	1
103	4	0.854	0.764	0.572	0.453	NA	2	2	2	1	1
104	4	0.739	0.769	0.489	0.409	NA	1	1	1	1	1
105	4	0.965	0.773	0.704	0.406	NA	2	2	2	2	1
106	4	0.849	0.684	0.870	0.453	NA	2	2	2	2	2
107	4	0.603	0.491	0.388	0.268	NA	1	1	1	2	1
108	4	0.903	0.480	0.556	0.659	NA	2	2	2	2	2
109	4	0.732	0.670	0.641	0.653	NA	1	2	2	2	1
110	4	0.783	0.748	0.719	0.926	NA	2	2	2	2	2
111	4	0.787	0.745	0.518	0.405	NA	1	2	1	1	1
112	4	0.822	0.694	0.716	0.392	NA	1	2	2	2	2
113	4	0.699	0.407	0.444	0.251	NA	1	1	1	1	1
114	4	0.703	0.660	0.532	0.329	NA	2	2	2	2	2
115	4	0.875	0.536	0.390	0.257	NA	1	2	2	2	2
116	4	1.157	0.928	0.790	0.607	NA	1	1	1	2	2
117	4	0.822	0.618	0.519	0.391	NA	1	1	1	1	2
118	4	0.669	0.483	0.418	0.217	NA	1	1	1	1	1
119	4	0.742	0.911	0.536	0.288	NA	1	2	2	2	2
120	4	0.865	1.054	0.863	0.588	NA	1	1	1	1	2
121	4	0.857	1.831	0.900	0.883	NA	2	1	1	1	2
122	4	0.679	0.590	0.325	0.333	NA	2	1	1	1	1
123	4	1.350	0.915	0.768	0.282	NA	1	1	1	1	2
124	4	0.684	0.524	0.507	0.287	NA	2	2	2	2	1
125	4	1.015	0.866	0.748	0.498	NA	2	1	1	1	2

126	4	1.662	0.815	0.677	0.198	NA	2	1	2	2	2
127	4	1.442	0.887	0.450	0.266	NA	1	1	1	1	2
128	8	0.879	0.763	0.680	0.345	0.116	2	2	2	2	1
129	8	0.789	0.566	0.422	0.467	0.351	1	2	2	2	2
130	4	0.625	0.638	0.599	0.522	NA	2	1	1	1	1
131	4	0.668	0.697	0.507	0.442	NA	1	2	2	2	2
132	8	0.756	0.889	1.224	0.827	0.124	2	2	2	2	1
133	4	0.849	0.701	0.506	0.414	NA	2	1	1	1	1
134	4	0.562	0.610	0.445	0.445	NA	1	1	1	1	1
135	4	0.694	0.541	0.557	0.391	NA	1	1	1	1	2
136	4	0.688	0.646	0.587	0.400	NA	1	2	2	2	1
137	8	0.643	0.513	0.491	0.521	0.007	2	2	2	2	2
138	8	0.700	0.522	0.414	0.286	0.006	2	1	1	1	2
139	4	0.661	0.517	0.439	0.439	NA	1	1	1	1	2
140	4	0.680	0.435	0.389	0.395	NA	1	2	2	2	2
141	4	0.667	0.513	0.469	0.403	NA	1	1	1	1	1
142	4	0.889	0.611	0.596	0.631	NA	1	2	2	2	2
143	4	0.853	0.716	0.456	0.386	NA	2	1	1	1	1

NA = Not applicable
