# Supplementary material for: Genetic Analyses of Amphotericin B Susceptibility in Aspergillus fumigatus
Source: J Fungi (Basel). 2021 Oct 14;7(10):860. doi: 10.3390/jof7100860 (PMC8538161; doi:10.3390/jof7100860)
Supplement: Supplementary file 1 [file jof-07-00860-s001.zip › Supplementary Table S3.pdf]

Chromosome	Position (bp)	Reference Allele (0)	Alternative Allele (1)	AFB62-1 Genotype	CM11 Genotype
7	278,099	A	G	0	0
5	2,362,267	G	A	0	1
5	2,386,509	T	G	0	0
4	3,275,045	T	A	0	0
2	4,385,926	G	A	0	0
1	3,787,543	A	G	0	0
5	3,698,701	G	T	0	0
6	1,608,813	C	T	0	1
3	1,260,557	T	C	1	0
6	3,521,360	G	A	0	0
6	3,141,751	G	A	0	0
6	3,149,653	G	T	0	0
3	133,642	T	C	0	0
3	142,183	A	C	0	0
7	1,182,007	A	C	0	0
7	279,416	T	C	0	0
4	2,417,511	A	G	1	1
4	2,417,525	G	T	0	0
3	3,512,400	T	C	1	0
3	3,122,663	A	C	0	0
4	2,416,428	A	G	1	1
4	2,417,416	A	C	1	1
4	2,417,517	T	C	1	1
4	2,417,806	C	T	1	1
5	201,094	T	C	0	1
5	201,751	G	A	0	1
5	2,370,937	C	G	0	1
5	2,399,121	G	A	0	1
